# *Ganoderma lucidum* reduces obesity in mice by modulating the composition of the gut microbiota

**DOI:** 10.1038/ncomms8489

**Published:** 2015-06-23

**Authors:** Chih-Jung Chang, Chuan-Sheng Lin, Chia-Chen Lu, Jan Martel, Yun-Fei Ko, David M. Ojcius, Shun-Fu Tseng, Tsung-Ru Wu, Yi-Yuan Margaret Chen, John D. Young, Hsin-Chih Lai

**Affiliations:** 1 Center for Molecular and Clinical Immunology, Chang Gung University, Gueishan, Taoyuan 33302, Taiwan, ROC; 2 Department of Medical Biotechnology and Laboratory Science, College of Medicine, Chang Gung University, Gueishan, Taoyuan 33302, Taiwan, ROC; 3 Department of Laboratory Medicine, Chang Gung Memorial Hospital, Linkou, Gueishan, Taoyuan 33305, Taiwan, ROC; 4 Department of Microbiology and Immunology, Chang Gung University, Gueishan, Taoyuan 33302, Taiwan, ROC; 5 Research Center of Bacterial Pathogenesis, Chang Gung University, Gueishan, Taoyuan 33302, Taiwan, ROC; 6 Department of Respiratory Therapy, Fu Jen Catholic University, Xinzhuang, New Taipei City 24205, Taiwan, ROC; 7 Chang Gung Biotechnology Corporation, Taipei 10508, Taiwan, ROC; 8 Biochemical Engineering Research Center, Ming Chi University of Technology, Taishan, New Taipei City 24301, Taiwan, ROC; 9 Department of Biomedical Sciences, University of the Pacific, Arthur Dugoni School of Dentistry, San Francisco, California 94103, USA; 10 Laboratory of Cellular Physiology and Immunology, Rockefeller University, New York, New York 10021, USA

## Abstract

Obesity is associated with low-grade chronic inflammation and intestinal dysbiosis. *Ganoderma lucidum* is a medicinal mushroom used in traditional Chinese medicine with putative anti-diabetic effects. Here, we show that a water extract of *Ganoderma lucidum* mycelium (WEGL) reduces body weight, inflammation and insulin resistance in mice fed a high-fat diet (HFD). Our data indicate that WEGL not only reverses HFD-induced gut dysbiosis—as indicated by the decreased Firmicutes-to-Bacteroidetes ratios and endotoxin-bearing Proteobacteria levels—but also maintains intestinal barrier integrity and reduces metabolic endotoxemia. The anti-obesity and microbiota-modulating effects are transmissible via horizontal faeces transfer from WEGL-treated mice to HFD-fed mice. We further show that high molecular weight polysaccharides (>300 kDa) isolated from the WEGL extract produce similar anti-obesity and microbiota-modulating effects. Our results indicate that *G. lucidum* and its high molecular weight polysaccharides may be used as prebiotic agents to prevent gut dysbiosis and obesity-related metabolic disorders in obese individuals.

## Abstract (web summary)

*Ganoderma lucidum* is a medicinal mushroom used in Traditional Chinese Medicine with putative anti-diabetic properties. Here, the authors show that polysaccharides from a water extract of this mushroom exert beneficial metabolic effects by modulating the composition of the gut microbiota in mice.

## Graphical abstract

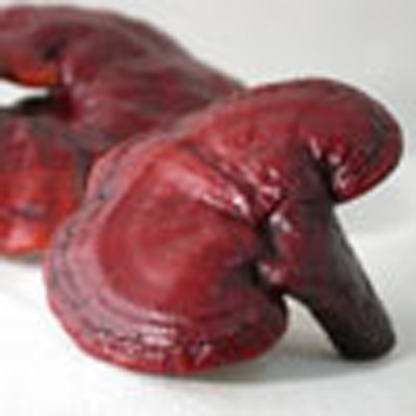

Traditional Chinese Medicine has a long history in Asian countries dating back several thousands of years^1^,^2^. One class of traditional remedies commonly in use consists of medicinal mushrooms such as *Ophiocordyceps sinensis*, *Antrodia cinnamomea* and *Agaricus blazei* Murrill, which contain a wide range of immuno-modulatory and bioactive compounds^3^,^4^. One of the most intriguing medicinal mushrooms is the *Basidiomycete* fungus *Ganoderma lucidum*, which has been used for centuries to promote health and longevity^5^. Previous studies have shown that triterpenes and polysaccharides isolated from *G. lucidum* inhibit adipocyte differentiation^6^ and produce hypoglycaemia effects in diabetic mice^7^. In addition, proteoglycans isolated from *G. lucidum* fruiting bodies induce antidiabetic, antihyperlipidemic and antioxidant activities^8^. However, it remained unknown whether *G. lucidum* produces any effect on body weight and obesity-related disorders.

Obesity is defined as a disease condition associated with numerous health problems and a reduced life expectancy^9^. Growing evidence indicates that obesity is closely linked with chronic, low-grade inflammation, which can lead to insulin resistance, type 2 diabetes, fatty liver disease, cardiovascular disease, obstructive sleep apnoea and cancer^10^,^11^. The high prevalence of obesity is currently a major threat to public health, with ∼500 million obese people and 1.4 billion overweight individuals worldwide^12^. Prevention of obesity thus represents a major challenge for modern societies.

A recent study indicates that changes in the composition of the gut microbiota are associated with the development of obesity and its associated metabolic disorders^13^. The gut microbiota comprises trillions of bacteria that contribute to nutrient acquisition and energy regulation^14^,^15^. An increased ratio of the major phyla Firmicutes/Bacteroidetes and changes in several bacterial species can promote the development of obesity in both dietary and genetic models of obesity in mice^16^,^17^. Other studies in obese animals suggest that obesity-induced gut dysbiosis caused by either environmental or genetic factors impairs intestinal integrity^18^,^19^. This process leads to the release of the endotoxin lipopolysaccaride (LPS) from intestinal Gram-negative bacteria into the bloodstream^20^, in turn, leading to metabolic inflammation and insulin resistance in obese mice^21^ due to stimulation of Toll-like receptor 4 (TLR4)-mediated inflammation^22^. Moreover, chronic injection of LPS in mice leads to mild obesity and insulin resistance^21^, highlighting a possible role for microbiota-derived LPS in obesity-induced inflammation.

A number of treatments, including antibiotics and prebiotics^18^,^19^, are being evaluated for the management of obesity and its related metabolic disorders^23^. For example, antibiotic treatment alters the gut microbiota, reduces blood endotoxemia and improves glucose tolerance in mice lacking the leptin gene (*ob/ob* mice) or in mice fed with a HFD^19^. In addition, prebiotics are non-digestible, fermentable carbohydrates and fibres, which reduce body weight and exert anti-inflammatory effects mainly by enhancing the growth of specific beneficial bacteria found in the gut^24^,^25^. Prebiotics not only alter the intestinal microbiota but also improve intestinal tight junction integrity and decrease blood endotoxemia caused by LPS^18^. Prebiotics may, therefore, protect animals against obesity-induced inflammation.

In the present study, we examined whether a water extract of *G. lucidum* mycelium (WEGL) can decrease obesity in HFD-fed mice. Our results indicate that WEGL reduces obesity and inflammation in the treated mice. These effects are transmissible to HFD-fed mice through horizontal faeces transplantation, indicating that the effects of WEGL involve the gut microbiota. Characterization of WEGL showed that polysaccharides of molecular weight >300 kDa exerted similar ameliorative effects as WEGL. These results implied that the high molecular weight polysaccharides may be the active components of WEGL. Our data thus demonstrate that WEGL represents a potential prebiotic agent that may be used for the treatment of obesity and its complications.

## Results

### WEGL prevents HFD-induced obesity in mice

**Figure 1:**
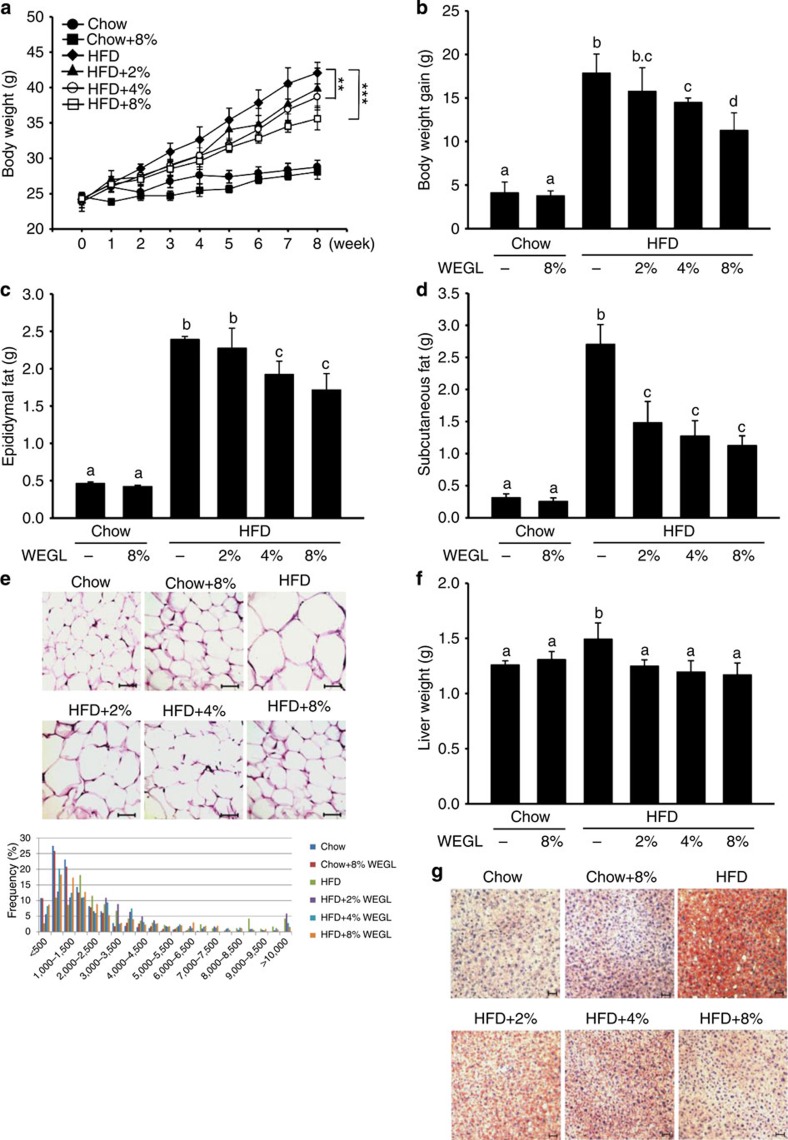
WEGL reduces body weight and fat accumulation in HFD-fed mice. Chow- and HFD-fed mice were treated daily with 100 μl of either water or WEGL at 2, 4 or 8% (w/v) by intragastric gavage for two months (*n*=7 for each group). Effects of WEGL treatment on body weight (**a**) body weight gain (**b**) epididymal fat (**c**) subcutaneous fat (**d**) and epididymal adipocyte size (**e**) are shown. In **e**, adipocyte size was estimated using the Image J software (lower panel). Scale bar, 50 μm. Liver weight was measured in HFD and control, chow-fed mice (**f**). Liver lipid content was assessed using oil red O staining (**g**). Scale bar, 30 μm. Data are expressed as mean±s.e.m. Body weight differences in **a** were analysed using unpaired two-tailed Student’s *t*-test (**P<0.01, ***P<0.001). Graph bars in **b, c, d** and **f** marked with different letters on top represent statistically significant results (*P*<0.05) based on Newman–Keuls *post hoc* one-way ANOVA analysis, whereas bars labelled with the same letter correspond to results that show no statistically significant differences. In the case where two letters are present on top of the bar in **b**, each letter should be compared separately with the letters of other bars to determine whether the results show statistically significant differences.

Using a mouse model of obesity, we observed that HFD feeding for 8 weeks led to significant increases in body and liver weight, epididymal and subcutaneous fat accumulation, and lipid deposition in adipocytes and hepatocytes compared with control chow feeding (Fig. 1a–g). While 8% WEGL did not produce any apparent effects in chow-fed mice, supplementation with WEGL decreased weight gain and fat accumulation in a dose-dependent manner in HFD-fed mice (Fig. 1a–g). Mean energy intake, stool fat and faeces energy did not vary significantly between HFD-fed groups (Supplementary Fig. 1), suggesting that the effects of WEGL on body weight and obesity parameters were not due to reduced food consumption or energy extraction. These results imply that WEGL reduces weight gain and fat accumulation in HFD-fed mice.

### WEGL reduces inflammation in HFD-fed mice

**Figure 2:**
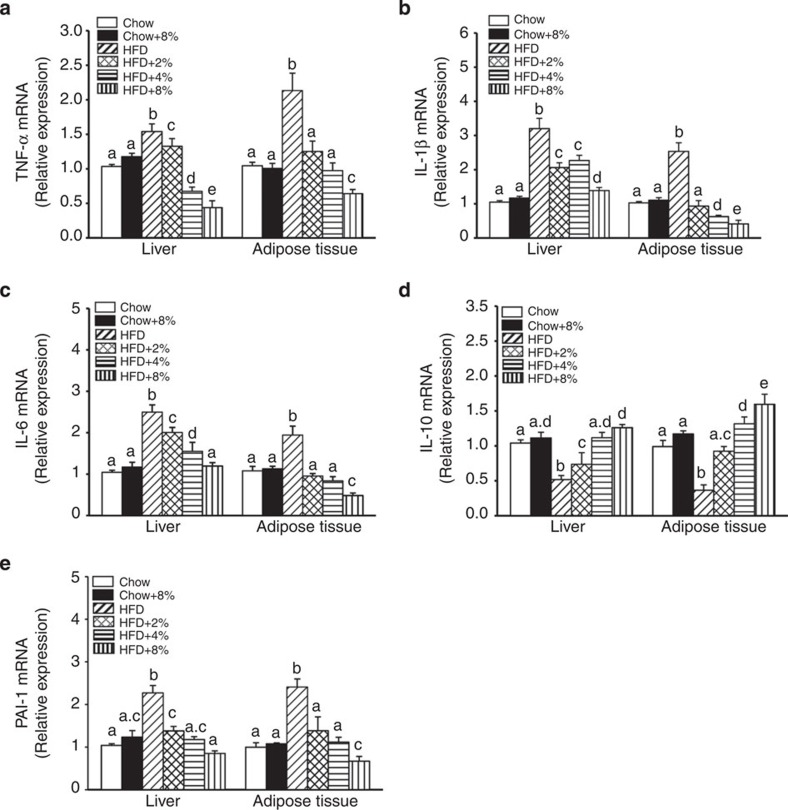
WEGL decreases pro-inflammatory cytokine expression in the liver and adipose tissues of HFD-fed mice. Animals were treated as in Fig. 1. Relative expression of TNF-α (**a**), IL-1β (**b**), IL-6 (**c**), IL-10 (**d**) and PAI-1 (**e**) in hepatic and adipose tissues was assessed using qRT–PCR and in comparison with the Chow group. Data are shown as mean±s.e.m. Graph bars with different letters on top represent statistically significant results (*P*<0.05) based on Newman–Keuls *post hoc* one-way ANOVA analysis, whereas bars with the same letter correspond to results that show no statistically significant differences. In the case where two letters are present on top of the bars in **d**,**e**, each letter should be compared separately with the letters of other bars to determine whether the results show statistically significant differences.

Previous studies have shown that HFD-fed obese mice produce higher levels of pro-inflammatory cytokines in hepatic and adipose tissues, including tumour necrosis factor-alpha (TNF-α), interleukin-1-beta (IL-1β), interleukin-6 (IL-6) and plasminogen activator inhibitor-1 (PAI-1; ref. 26). In contrast, production of the anti-inflammatory cytokine IL-10 is reduced in obese animals^27^. We measured messenger RNA (mRNA) expression of these cytokines after 8 weeks of HFD feeding with or without WEGL supplementation. TNF-α, IL-1β, IL-6 and PAI-1 expression levels were higher in hepatic and adipose tissues of HFD-fed mice compared with tissues of control chow-fed mice, whereas IL-10 expression was reduced (Fig. 2a–e). Notably, the expression pattern of these cytokines was altered in a dose-dependent manner by WEGL treatment, resulting in expression levels closer to that of chow-fed mice than HFD-fed mice with increasing WEGL dose (Fig. 2a–e). Moreover, WEGL reduced the levels of secreted TNF-α, IL-1β and IL-6 proteins in a dose-dependent manner in the serum of HFD mice (Supplementary Fig. 2).

Obesity is characterized by infiltration and activation of immune cells in hepatic and adipose tissues^28^. M1 macrophages, which are recruited by monocyte chemoattractant protein-1 (MCP-1), are associated with chronic, low-grade inflammation in adipose tissues of obese animals^26^. As MCP-1 mRNA expression decreased in the liver and adipose tissues of HFD-fed mice following treatment with WEGL (Supplementary Fig. 3a), we examined the numbers of macrophages recruited into hepatic and adipose tissues using flow cytometry analysis. Double staining of macrophages with anti-F4/80 antibody combined with either anti-CD11b antibody (for liver macrophages, also called Kupffer cells) or anti-CD11c antibody (for macrophages in adipose tissues) was used for these experiments. Higher levels of macrophages were detected in hepatic and adipose tissues of HFD mice compared with chow-fed mice (Supplementary Fig. 3b,c). While macrophage levels increased following the treatment with 8% WEGL in chow-fed mice, these cells were reduced in a dose-dependent manner by WEGL in hepatic and adipose tissues of HFD-fed mice (Supplementary Fig. 3b,c).

Previous studies indicated that HFD feeding also produces a gradual loss of regulatory T (Treg) cells compared with chow-fed mice^26^. While HFD also reduced Treg levels in our mouse model, supplementation with WEGL led to dose-dependent increases of Treg cells in liver and adipose tissues of HFD mice (Supplementary Fig. 3d,e). In addition, chow-fed mice treated with 8% WEGL showed increased Treg levels compared with untreated chow-fed mice (Supplementary Fig. 3d,e). These results indicate that WEGL reduces inflammation in HFD-fed mice by reducing macrophage infiltration and enhancing Treg accumulation in hepatic and adipose tissues.

### WEGL reduces endotoxemia and insulin resistance in HFD mice

**Figure 3:**
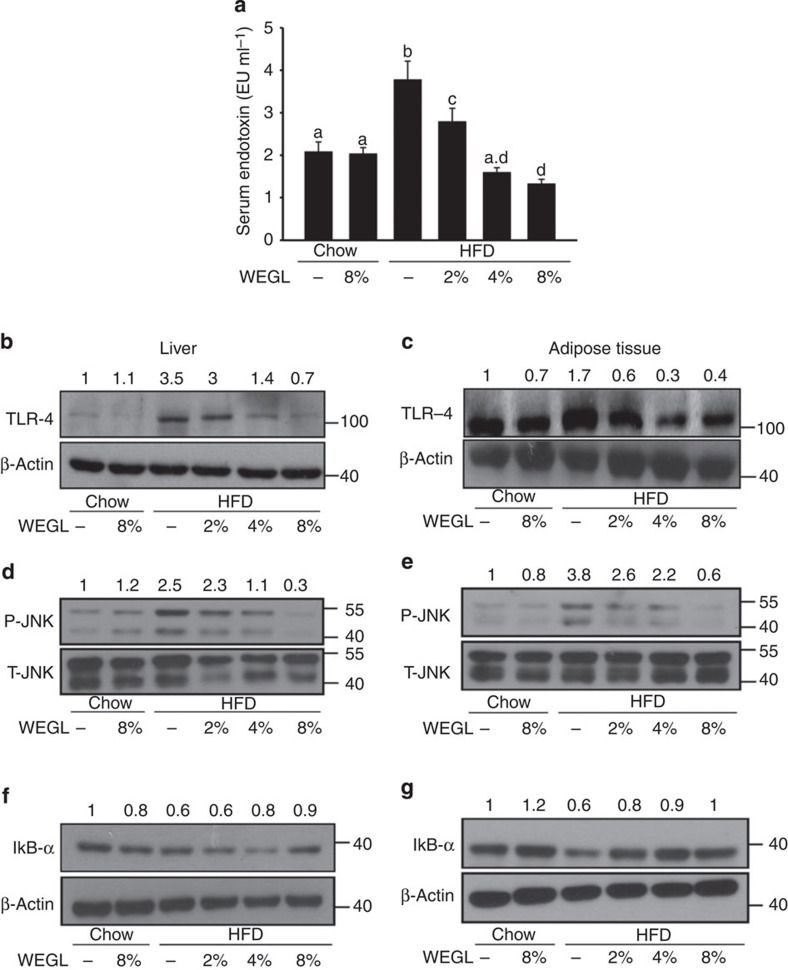
WEGL reduces serum LPS and TLR4-related signalling pathways in HFD mice. Effects of WEGL treatment on serum endotoxin (**a**) TLR4 protein production (**b**,**c**) JNK phosphorylation (**d**,**e**) and IκB-α production (**f**,**g**) were examined in the liver and epididymal adipose tissues of chow- and HFD-fed mice as described in Fig. 1. Serum endotoxin (EU ml^−1^) was determined as mean±s.e.m. using the limulus amebocyte lysate assay kit. Representative immunoblots for target proteins in **b-g** are shown. Molecular weight markers were indicated as kilodaltons (kDa). Protein levels were normalized to internal controls (β-actin or total JNK, T-JNK) and the relative ratio to the Chow group was labelled on the top of immunoblots. Graph bars in **a** with different letters on top represent statistically significant results (*P*<0.05) based on Newman–Keuls *post hoc* one-way ANOVA analysis, whereas bars labelled with the same letter correspond to results that show no statistically significant differences. Where two letters are present on top of the bar, each letter should be compared separately with the letters of other bars to determine whether the results show statistically significant differences.

Endotoxemia and TLR4 signalling control the production of pro-inflammatory cytokines in target tissues and lead to chronic inflammation and insulin resistance in HFD-fed mice^19^. We examined the effects of WEGL on LPS serum levels (that is, endotoxemia) and TLR4 protein expression in hepatic and adipose tissues. WEGL reduced endotoxemia and TLR4 protein expression in HFD-fed mice compared with HFD alone (Fig. 3a–c). Since TLR4 signalling pathways induce the production of pro-inflammatory cytokines by modulating the activity of JNK and NF-κB^29^,^30^, we examined whether these pathways are affected by WEGL supplementation. As shown in Fig. 3d,e, WEGL inhibited JNK phosphorylation in hepatic and adipose tissues of HFD-fed mice. Moreover, the production of IκB-α, whose interaction with NF-κB prevents NF-κB translocation and activation, was enhanced by WEGL treatment (Fig. 3f,g).

Given that enhanced activation of JNK and NF-κB pathways may induce insulin resistance via phosphorylation of insulin receptor substrate-1 (IRS-1) on serine 307 and dephosphorylation of Akt^31^, we examined the effects of WEGL on insulin activity. As shown in Supplementary Fig. 4a–e, WEGL treatment reduced fasting insulin and glucose levels and decreased glucose and insulin resistance in HFD-fed mice. Accordingly, phosphorylation of serine 307 on IRS-1 was inhibited by WEGL in hepatic and adipose tissues, whereas phosphorylation of Akt was enhanced by the mycelium extract (Supplementary Fig. 4f–i). WEGL therefore reduces endotoxemia and prevents insulin resistance in HFD-fed mice.

### WEGL regulates lipogenic gene expression

Previous studies have shown that the expression of genes involved in lipid biosynthesis such as acetyl-CoA carboxylase-1, fatty acid synthase, sterol regulatory element-binding protein-1 and peroxisome proliferator-activated receptors-γ is enhanced in hepatic and adipose tissues of HFD-fed mice^32^,^33^. Our results indicated that 8% WEGL did not significantly affect the expression of these genes in chow-fed mice (Supplementary Fig. 5a–d). In contrast, WEGL reduced lipogenic gene expression in a dose-dependent manner in HFD-fed mice (Supplementary Fig. 5a–d).

Increased levels of free fatty acids (FFA) in the blood of obese animals are thought to arise from a larger mass of adipose tissue and enhanced inflammation in HFD-fed mice^34^. These circulating fatty acids also induce TLR4 signalling in target tissues and cause insulin resistance^22^. We observed that WEGL reduced serum FFA levels in HFD mice (Supplementary Fig. 5e). WEGL may thus reduce fat accumulation by inhibiting adipogenic gene expression and decreasing serum FFA in HFD-fed mice.

### WEGL reverses HFD-induced gut dysbiosis

The gut microbiota of obese humans and HFD-fed mice is characterized by an increased Firmicutes-to-Bacteroidetes ratio, elevated endotoxin-producing Proteobacteria, and reduced immuno-homeostatic bacterial species^35^,^36^. We examined the effects of WEGL on gut microbiota composition by performing a pyrosequencing-based analysis of bacterial 16S rRNA (V3–V5 region) in caecal faeces. After removing unqualified sequences (see Methods), a total of 691,370 raw reads and an average of 16,461±5,411 reads per sample were obtained. After selecting the effective reads, a total of 292,952 effective reads was generated and each faecal sample (*n*=7 for each group) produced an average of 6,975±2,192 effective reads. Samples with a low number of effective reads (<3,000) were not observed. Rarefaction and Shannon index analyses indicated that the sequencing depth covered rare new phylotypes and most of the diversity (Supplementary Fig. 6).

**Figure 4:**
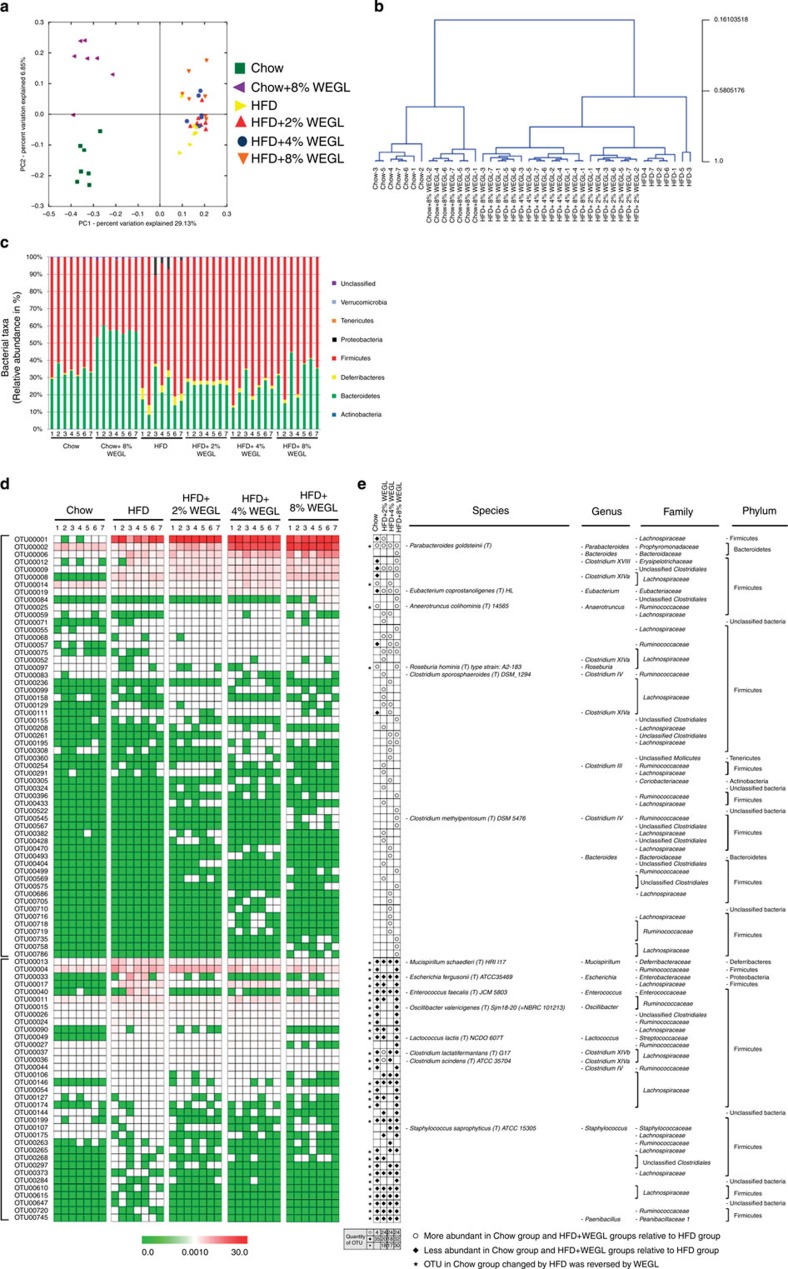
WEGL alters microbiota composition in HFD-fed mice. Microbiota composition in faeces of chow-fed mice treated with or without 8% WEGL and HFD mice treated with 2, 4 or 8% WEGL were analysed using next generation sequencing (*n*=7 for each group). (**a**) Plots shown were generated using the weighted version of the UniFrac-based PCoA. (**b**) Multivariate analysis of variance from PCoA matrix scores. (**c**) Bacterial taxonomic profiling in the phylum level of intestinal bacteria from different mouse groups. (**d**) Heatmap showing the abundance of 91 OTUs significantly altered by WEGL in HFD-fed mice based on RDA. (**e**) Represented bacterial taxa information (species, genus, family and phylum) of 91 OTUs from **d** are shown. White circles and black diamonds indicate the OTUs that increased or decreased in Chow- and HFD+WEGL-fed groups relative to the HFD-fed group. Black stars represent OTUs whose abundance in chow-fed mice was altered by HFD and then reversed by WEGL. OTU taxonomy is shown on the right.

UniFrac-based principal coordinates analysis (PCoA) revealed a distinct clustering of microbiota composition for each treatment group (Fig. 4a). Multivariate analysis of variance of PCoA matrix scores indicated a statistically significant separation between the microbiota of Chow and Chow+8% WEGL groups (Fig. 4b). Significant separations were also noted for HFD, HFD+4% WEGL and HFD+8% WEGL groups (Fig. 4b). On the other hand, the gut microbiota of HFD+2% WEGL mice did not significantly differ from that of HFD mice (Fig. 4b), consistent with the mild effects produced by 2% WEGL (Figs 1, 2, 3). Notably, taxonomic profiling demonstrated that treatment with 4% and 8% WEGL reduced the ratio of Firmicutes to Bacteroidetes and the Proteobacteria phylum in HFD-fed mice to levels similar to that of chow-fed mice (Fig. 4c). Here as well, the changes produced by the 2% WEGL treatment were not observed (Fig. 4c).

We used redundancy analysis (RDA) to identify the specific bacterial phylotypes that were altered by HFD feeding and WEGL treatment. Compared with chow-fed mice, HFD feeding significantly altered 209 operational taxonomic units (OTUs), producing 44 increased and 165 decreased OTUs (Supplementary Data 1 and Supplementary Fig. 7a). In HFD-fed mice, supplementation with 2, 4 and 8% WEGL, respectively, altered 44 (24 increased and 20 decreased), 42 (24 increased and 18 decreased) and 56 OTUs (24 increased and 32 decreased; Supplementary Data 2 and Supplementary Fig. 7b–d), resulting in significant changes in a total of 91 distinct OTUs (Fig. 4d). Among the 91 OTUs that were altered compared with HFD-fed mice, 2, 4 and 8% WEGL treatments altered OTUs in the same direction in Chow mice for 18, 17 and 30 OTUs, respectively (Fig. 4e, highlighted with black stars). These results suggest that 8% WEGL is the most effective treatment for modulating the gut microbiota in our model.

Detailed analysis of the 30 OTUs reversed by 8% WEGL indicated that *Mucispirilum shaedleri*^37^, *Escherichia fergusonii* (Proteobacteria^20^), *Enterococcus* spp.^38^, *Lactococcus lactis*^39^, *Clostridium lactatifermentans* (Clostridium XIVb) and *Oscillibacter valericigenes*^25^,^40^ (which were found to be enhanced by HFD and to positively correlate with obesity in the previous studies cited above) were all reversed by 8% WEGL (Fig. 4d,e). Notably, in comparison with HFD mice, 8% WEGL treatment enhanced a variety of bacterial species that negatively correlate with obesity, including *Parabacteroides goldsteinii*, *Bacteroides* spp., *Anaerotruncus colihominis*, *Roseburia hominis*, *Clostridium methylpentosum* (Clostridium IV), Clostridium XIVa and XVIII and *Eubacterium coprostanoligenes* (Fig. 4d,e and Supplementary Data 2; note that these species were initially reduced by HFD compared with chow feeding). Bacterial species including *E. coprostanoligenes*, *C. methylpentosum*, *P. goldsteinii*, *Bacteroides* spp., *A*. *colihominis*, *R*. *hominis* and *Clostridium* XIVa and XVIII were also enriched in the Chow+8% WEGL treatment compared with the chow diet (Supplementary Data 3 and Supplementary Fig. 7e). Moreover, many bacterial species increased in the 8% WEGL group but were not altered by HFD, indicating that WEGL may enrich specific bacterial species (Fig. 4e and Supplementary Data 2). Collectively, these results show that WEGL modulates the gut microbiota of HFD-fed mice, resulting in a microbiota composition similar to that of chow-fed mice.

### WEGL maintains intestinal integrity in HFD mice

Given that intestinal dysbiosis in HFD-fed animals may affect gut permeability and subsequently lead to release of bacterial LPS into the circulation^19^, we examined whether WEGL modulates gut integrity. While HFD feeding reduced expression of the tight junction components, zonula occludens-1 (ZO-1) and occludin, these effects were reversed by WEGL supplementation (Supplementary Fig. 8). These findings suggest that WEGL may improve intestinal barrier integrity in HFD-fed mice.

### WEGL faecal transplants reduce obesity

**Figure 5:**
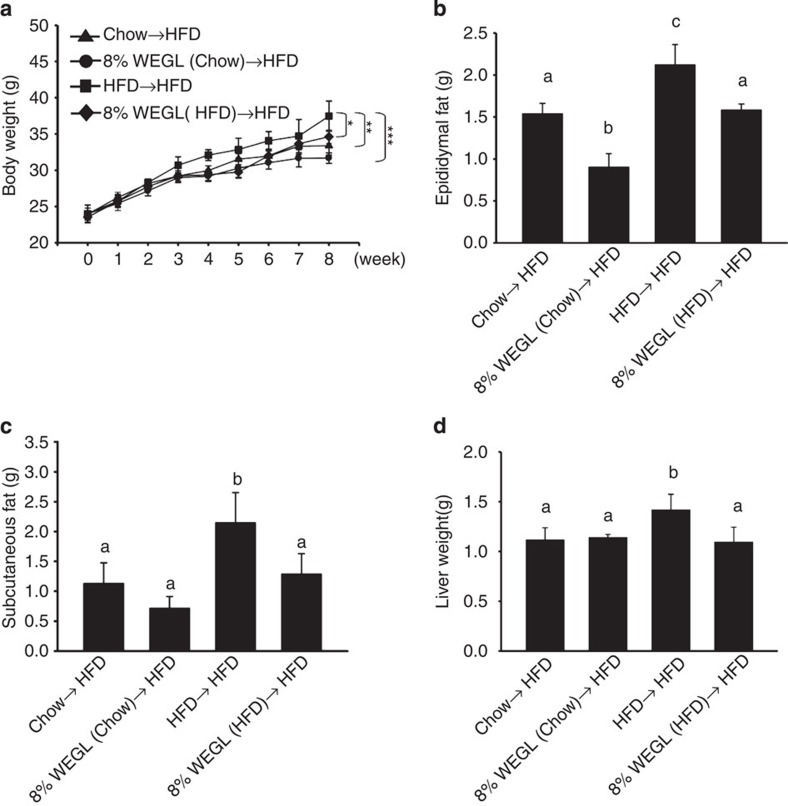
Obesity and fat accumulation are reversed by faecal transplantation from WEGL-treated mice to HFD-fed mice. Eight-week-old HFD-fed mice were colonized with faeces from different mouse groups for 8 weeks, followed by measurement of body weight (**a**) epididymal fat (**b**) subcutaneous fat (**c**) and liver weight (**d**). Each group consisted of five mice. Body weight differences in **a** were analysed using unpaired two-tailed Student’s *t*-test (*P<0.05, **P<0.01, ***P<0.001). Graph bars in **b–d** with different letters on top represent statistically significant results (*P*<0.05) based on Newman–Keuls *post hoc* one-way ANOVA analysis, whereas data labelled with the same letter correspond to results that show no statistically significant differences.

Recent studies have shown that the ability of the gut microbiota to modulate obesity can be transferred to other animals^13^. To determine whether the gut microbiota of WEGL-treated animals may improve the condition of HFD-fed mice, we transferred the microbiota of WEGL-treated mice to HFD-fed mice, followed by examination of obesity-related traits. Analysis of the cohort of donor mice indicated that WEGL treatment reduced body weight and the Firmicutes-to-Bacteroidetes ratio in the gut microbiota of HFD-fed mice (Supplementary Fig. 9a,b), similar to the results shown above (Figs 1a and 4c). Our results further showed that horizontal faecal transfer from mice fed with chow (Chow→HFD), Chow+8% WEGL (8% WEGL (Chow)→HFD), or HFD+8% WEGL (8% WEGL (HFD)→HFD) reduced body weight, epididymal and subcutaneous fat accumulation and liver weight compared with faecal transfer from HFD-fed mice (HFD→HFD; Fig. 5a–d). Faecal transfer from the Chow+8% WEGL group produced the most robust effects on weight gain and fat accumulation (Fig. 5a–c).

**Figure 6:**
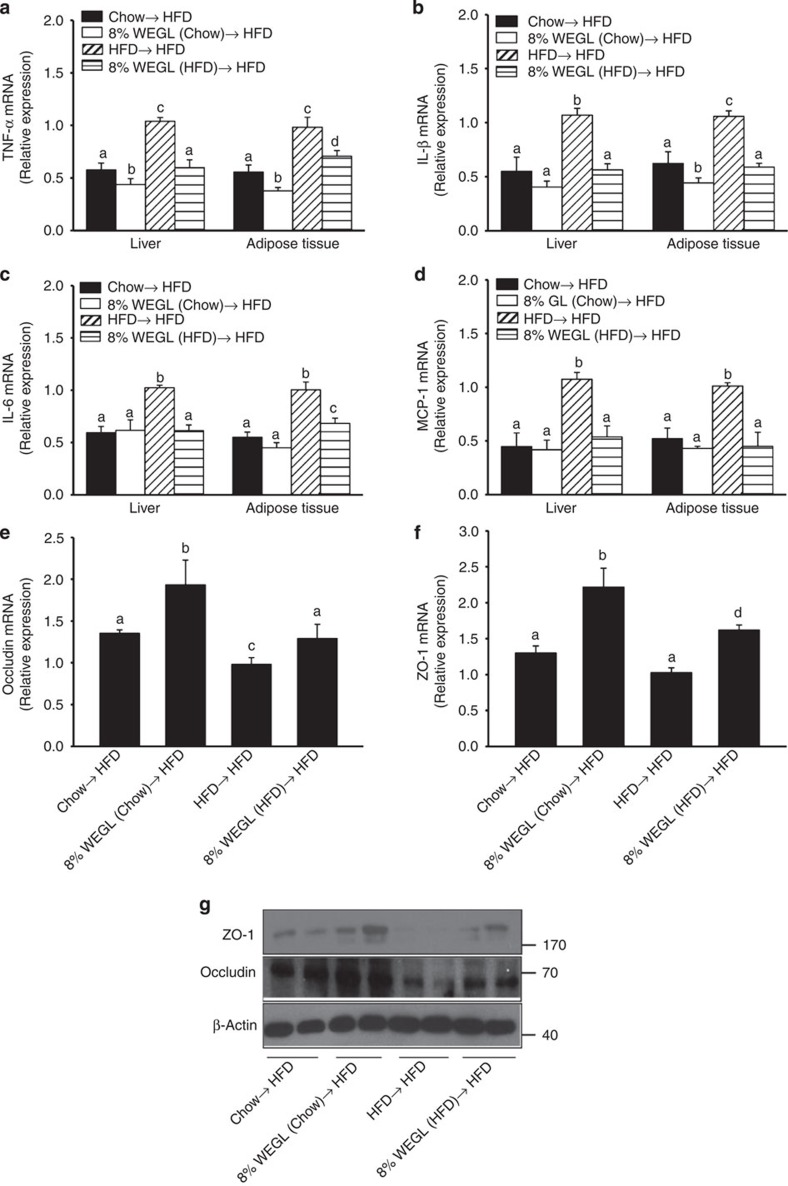
Analysis of pro-inflammatory cytokines and intestinal tight junctions following faecal transplantation from WEGL-treated mice. Eight-week-old HFD mice were colonized with faeces from the indicated mouse groups for 8 weeks. In comparison with the HFD→HFD group, relative mRNA expression levels of TNF-α (**a**) IL-1β (**b**) IL-6 (**c**) and MCP-1 (**d**) in hepatic and adipose tissues as well as occludin (**e**) and ZO-1 (**f**) in ileum, were assessed using qRT–PCR. Representative ileum immunoblots for occludin, ZO-1 and β-actin in each group. (**g**) Molecular weight markers were indicated as kDa. Each group consisted of five mice. Graph bars in **a–f** with different letters on top represent statistically significant results (*P*<0.05) based on Newman–Keuls *post hoc* one-way ANOVA analysis, whereas bars labelled with the same letter correspond to results with no statistically significant differences.

Expression of pro-inflammatory cytokines and lipogenic genes was also reduced in hepatic and adipose tissues of HFD mice after transfer of faeces derived from WEGL-treated animals (Fig. 6a–d and Supplementary Fig. 10). Furthermore, the mRNA and protein expression levels of tight junction proteins in the ileum segment were also increased following the transfer of faeces from WEGL-treated mice (Fig. 6e–g). Transfer of faeces from 8% WEGL-chow-fed mice to HFD mice also produced statistically significant changes in TNF-α expression (Fig. 6a), lipogenic genes (Supplementary Fig. 10) and ileum tight junctions (Fig. 6e–g), compared with faecal transfer from chow-fed mice. These results suggest that the weight-lowering effects of WEGL in HFD-fed mice may be due to modulation of the gut microbiota.

### WEGL faecal transplants modulate gut microbiota composition

**Figure 7:**
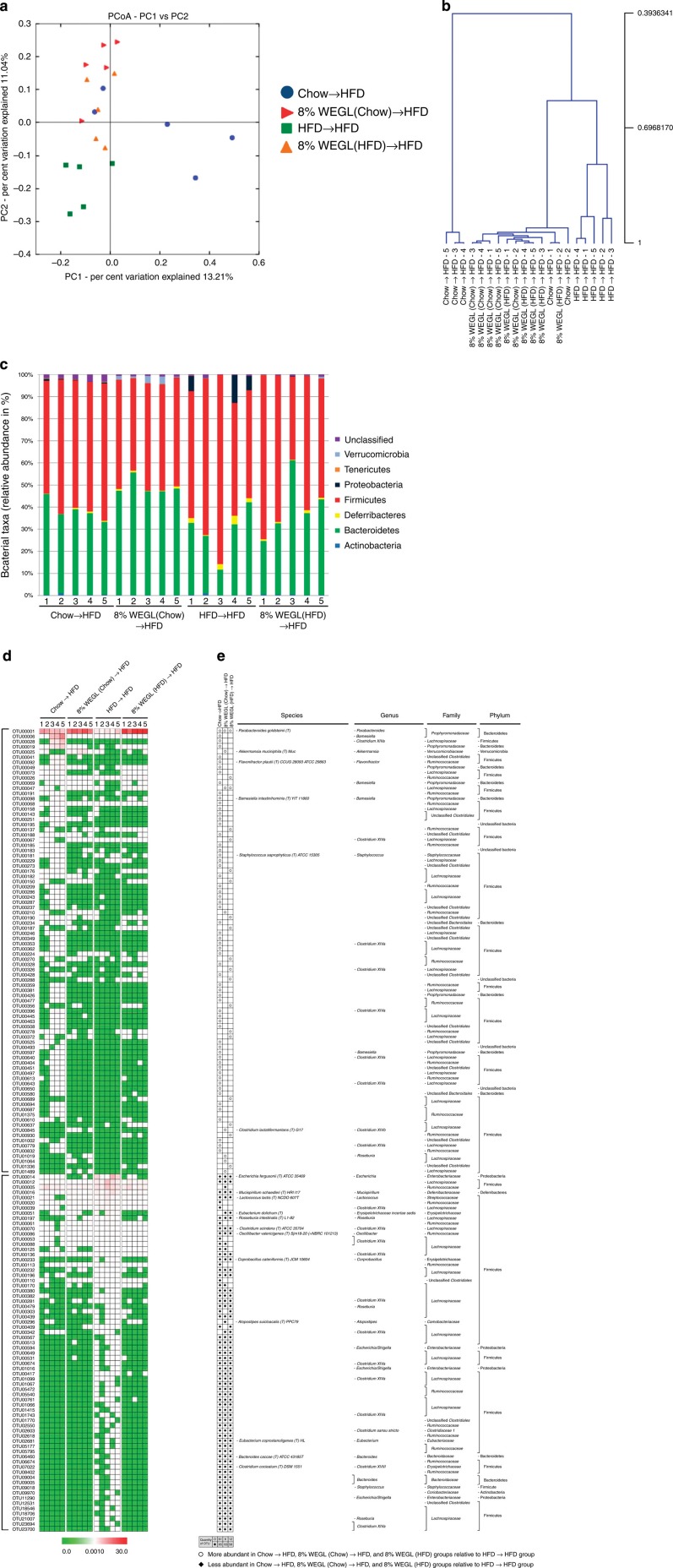
Analysis of gut microbiota following faecal transplantation. Faecal transplantation from Chow-, HFD- and Chow/HFD+8% WEGL-fed mice was performed and relevant microbiota analysis was done as described in the Methods. (**a**) The plots shown were generated using the weighted version of UniFrac-based PCoA. (**b**) Multivariate analysis of variance from PCoA matrix scores. (**c**) Bacterial taxonomic analysis of intestinal bacterium from each mouse groups. (**d**) Heatmap showing the abundance of 155 OTUs significantly altered by Chow, Chow+8% WEGL and HFD+8% WEGL transplanted mice based on RDA analysis. (**e**) Represented bacterial taxa information (species, genus, family and phylum) of 155 OTUs from **d** are shown. White circles and black diamonds indicate the OTUs that increased or decreased compared with HFD recipient mice.

In order to confirm that WEGL modulates the gut microbiota, we examined the composition of intestinal bacteria following faecal transfer from 8% WEGL-treated mice. In a separate sequencing analysis, a total of 1,056,611 raw reads and an average of 52,831±6,766 reads per sample were obtained. Subsequently, a total of 458,093 effective reads was generated and each faecal sample (*n*=5 for each group) produced 22,905±3,039 effective reads. Under this sequencing depth, rare phylotypes and most diversity were also covered (Supplementary Fig. 11). In comparison with mice that received faeces from HFD-fed mice, recipient HFD-fed mice (*n*=5 for each group) showed distinct microbiota after faecal transplantation from Chow-, Chow+8% WEGL- or HFD+8% WEGL-fed mice (Fig. 7a,b). Faecal transfer from HFD mice produced Firmicutes to Bacteroidetes ratios and Proteobacteria and Deferribacteres phyla levels that did not differ from those of HFD-fed mice (Fig. 7c, compared with Fig. 4c). In contrast, faeces transfer from Chow-, Chow+8% WEGL and HFD+8% WEGL-fed mice produced values similar to those of chow-fed or WEGL-treated mice (Fig. 7c, compared with Fig. 4c).

RDA analysis identified a total of 155 OTUs that were significantly altered by faecal transplant from Chow- (126 OTUs), Chow+8% WEGL- (71 OTUs) and HFD+8% WEGL-fed groups (75 OTUs) compared with HFD→HFD mice (Supplementary Data 4 and Supplementary Fig. 12). Among these, *M*. *shaedleri*, *E*. *fergusonii*, *L*. *lactis*, *C*. *scindens* and *O*. *valericigenes*, which were enhanced by HFD feeding (Fig. 4d), were all reversed by faecal transfer from Chow-, Chow+8% WEGL- and HFD+8% WEGL-fed mice (Fig. 7d,e). In addition, *Clostridium cocleatum*, *Bacteroides caccae, Eubacterium dolichum* and *Coprobacillus cateniformis* were altered after faecal transplantation from the same three groups of mice (Fig. 7d,e, these bacteria were not identified in the WEGL treatment experiments shown in Fig. 4). Of note, while the HFD-induced reduction of *P*. *goldsteinii* (Fig. 4d,e) was observed in HFD→HFD mice, this species was induced by faecal transplantation from the other three groups of mice (Fig. 7d,e). Interestingly, *Akkermansia muciniphila*, a potentially beneficial bacterial species, was only identified in HFD-fed mice that received faeces from Chow+8% WEGL-treated mice (Fig. 7d,e). These results indicate that WEGL restores the gut microbiota of HFD mice to a composition similar to those of chow-fed mice.

### WEGL high molecular weight polysaccharides reduce obesity

**Figure 8:**
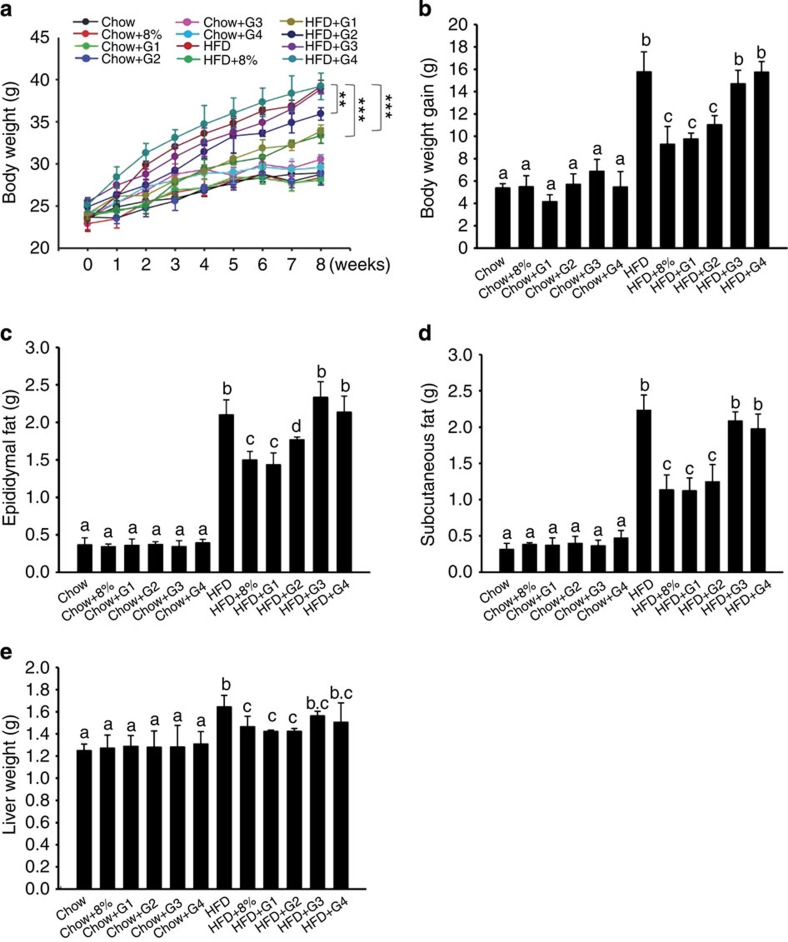
Effect of WEGL polysaccharide fractions on body weight and fat accumulation in HFD-fed mice. Mice fed with chow or HFD were treated daily with 100 μl of polysaccharide subfractions (G1, G2, G3, G4), 8% WEGL or water by intragastric gavage for 2 months (*n*=5 for each group). Effects of polysaccharide subfractions on body weight (**a**) body weight gain (**b**) epididymal fat (**c**) subcutaneous fat (**d**) and liver weight (**e**). Body weight differences in **a** were analysed using unpaired two-tailed Student’s *t*-test (**P<0.01, ***P<0.001). Graph bars in **b**–**e** labelled with different letters on top represent statistically significant results (*P*<0.05) based on Newman–Keuls *post hoc* one-way ANOVA analysis, whereas bars with the same letter correspond to results that show no statistically significant differences. In the case where two letters are present on top of the bars in **e**, each letter should be compared separately with the letters of the other bars to determine whether the results show statistically significant differences.

To identify the active ingredients of WEGL responsible for the anti-obesity effects, we fractionated the mycelium extract into four fractions (G1, G2, G3 and G4) on the basis of their molecular weights (Table 1). As shown in Fig. 8a–e, fraction G1, which consisted of polysaccharides with molecular weight >300 kDa (Table 1) and a monosaccharide composition comprising 47.5% mannose, 26.3% glucose and 16.9% galactose (Table 2), produced significant anti-obesity effects on body weight, liver weight and epididymal and subcutaneous fat in HFD-fed mice. Notably, the extent of anti-obesity effects observed was similar to that produced by the whole WEGL mycelium extract (Fig. 8a–e). In contrast, fraction G2 showed modest anti-obesity effects, whereas no significant effect was observed for fractions G3 and G4 (Fig. 8a–e). No significant change in mean energy intake, stool fat or energy extraction was noted following treatment with the polysaccharide fractions (Supplementary Fig. 13). These results suggest that the anti-obesity effects of WEGL mycelium may be due mainly to its high molecular weight polysaccharide fraction.

## Discussion

Although previous studies have shown that *G*. *lucidum* lowers serum glucose and produces beneficial effects on type 2 diabetes mellitus in murine models^6^,^41^,^42^, the effects of this fungus on the gut microbiota, inflammation and obesity had not been investigated. Our study shows that *G*. *lucidum* mycelium prevents dietary-induced obesity and alleviates inflammation by modulating the composition of the gut microbiota and maintaining intestinal barrier integrity. Our results thus reveal that WEGL modulates the intestinal microbiota previously reported to correlate with obesity^43^, but also identify the high molecular weight polysaccharides (>300 kDa) as the major bioactive ingredients responsible for these effects.

Our observations that WEGL produces significant changes in the gut microbiota and the anti-obesity effects of WEGL are transferrable through faecal transplantation support the concept that obesity is associated with an altered gut microbiota. These findings are consistent with the results of a recent study by Ridaura *et al.*^13^ Using twins discordant for obesity as a model, these authors observed that transfer of the gut microbiota of obese individuals to HFD-fed mice conveyed larger weight gain and higher levels of obesity-associated metabolic disorders than faecal transfer from the individual’s lean twin. Our results suggest that the gut microbiota can be modulated by dietary intervention or faecal transfer, and that the WEGL mycelium extract may be used as prebiotics to produce a specific gut microbiota associated with reduced weight gain, inflammation and insulin resistance in obese individuals.

The current model of HFD-induced chronic inflammation and obesity-related disorders is explained largely by dysbiosis of the gut microbiota and increased levels of LPS in the blood, a condition called metabolic endotoxemia^19^. The intestinal lumen is a reservoir of LPS from Gram-negative bacteria that include *Escherichia* spp.^20^ In a state of intestinal dysbiosis as seen in HFD-fed animals, the intestinal tube may gradually become leaky, allowing the LPS of Gram-negative bacteria to enter the entero-hepatic circulation. Low concentrations of LPS in blood may cause systemic and targeted inflammation in HFD-fed mice and obese humans through activation of TLR4 signalling in various cells^22^. Our results indicate that WEGL supplementation improves gut barrier integrity, reduces endotoxemia, decreases TLR4 signalling and decreases inflammation in obese mice fed with a HFD. The beneficial effects induced by WEGL treatment may therefore be attributed to specific alterations in the gut microbiota (for example, reduction of *Escherichia* spp. such as *E. fergusonii*) and to maintenance of gut barrier integrity.

Macrophages found within adipose tissues exhibit two different activation profiles consisting of the M1 and M2 types^44^. LPS-induced M1 macrophages accumulate around dying adipocytes and hepatocytes and secrete pro-inflammatory cytokines, providing a mechanism to explain how obesity may propagate inflammation^45^. We observed that WEGL treatment reduces the number of macrophages and decreases MCP-1 expression in hepatic and adipose tissues of HFD-fed mice (Supplementary Fig. 3a–c). Furthermore, an increase of IL-10 expression (Fig. 2d) and Treg cells in liver and adipose tissues (Supplementary Fig. 3d,e) was also observed in WEGL-treated mice. In addition to macrophages and Treg cells, other immune cells such as NKT and B cells^46^,^47^,^48^ may also contribute to the anti-obesity effects observed here.

The gut microbiota of obese humans and animals is associated with decreased levels of intestinal Bacteroidetes and increased levels of Firmicutes, indicating that these major phyla may play a role in obesity-related inflammation^16^,^49^. Accordingly, prebiotics feeding with the plant hemicellulose compound arabinoxylan reverses the Bacteroides-to-Firmicutes ratio and reduces obesity^50^. We observed that 8% WEGL supplementation in HFD-fed mice restored the Firmicutes to Bacteroidetes ratio to that observed in chow-fed mice (Fig. 4c). Intriguingly, the probiotic *Bifidobacterium* spp., which were previously reported to reduce obesity^51^,^52^, were not detected in the present study. This observation suggests that WEGL may produce anti-obesity effects by altering the Firmicutes-to-Bacteroidetes ratio as well as modifying the levels of other specific bacterial species (Supplementary Data 2 and 3).

A previous study showed that chitin-glucan fibres modulate *Clostridium* cluster XIVa (*Roseburia* spp.) in the gut microbiota of HFD-fed mice^53^. Bacteria belonging to *Clostridium* clusters XIVa, XVIII and IV, which lack prominent toxins and virulence factors, were found earlier to modulate host fatty acid metabolism, induce Treg cell activity and attenuate colitis^54^. Furthermore, *Eubacterium* spp. induced by prebiotic oligosaccharides produce beneficial effects on animal hosts^55^, highlighting the potential probiotic effect of these species. Our results demonstrate that WEGL supplementation enhances bacterial levels of *Clostridium* clusters IV, XVIII and XIVa (*Roseburia* spp.), and *Eubacterium* spp. in HFD-fed obese mice (Fig. 4d,e and Supplementary Data 2). These results indicate that the effects of WEGL may be at least partially due to an increase in the populations of these beneficial species. WEGL feeding also decreased several bacterial species associated with inflammation and obesity. For instance, *E. fergusonii*, which is associated with HFD-induced inflammation^20^, was reduced following WEGL treatment (Fig. 4d,e and Supplementary Data 2). *Oscillibacter* spp. were also reported to increase in HFD-fed mice compared with chow-fed mice, and these bacteria showed a negative relationship with the expression of intestinal tight junction proteins^40^. Consistent with these observations, the 8% WEGL treatment reversed the percentage of *Oscillibacter* spp. in the gut microbiota of HFD-fed mice to a percentage similar to that seen in chow-fed mice (Fig. 4d,e and Supplementary Data 1 and 2). In addition, *Mucispirillum* spp. belonging to Deferribacteres, which are known to colonize the mucus layer, increased in HFD-fed mice compared with chow-fed mice^56^. In contrast, *Mucispirillum* spp. were reduced by WEGL in HFD mice in the present study (Fig. 4d,e and Supplementary Data 2). Therefore, WEGL may prevent HFD-induced obesity by modulating the composition of the gut microbiota in multiple ways^57^. Nonetheless, the hypothesis that WEGL may also directly affect pro-inflammatory signalling pathways such as TLR2, which also controls adiposity and insulin resistance^58^, cannot be ruled out.

In addition to modulation of the microbiota composition, our *in vivo* experiments demonstrate that the administration of WEGL also reduces expression of genes involved in fatty acid synthesis and reduces insulin resistance in HFD-fed mice. These results suggest that the effects of WEGL may be due to modulation of lipogenic gene expression. Moreover, a previous study demonstrated that over-production of pro-inflammatory cytokines such as TNF-α, IL-1β, IL-6 and MCP-1 in obese animals may be responsible for the development of chronic inflammation and insulin resistance, due at least in part to IRS-1 phosphorylation (on serine 307; ref. 31). In the present study, WEGL treatment induced the de-phosporylation of IRS-1 and enhanced phosphorylation of Akt, leading to improved insulin sensitivity. Thus, WEGL may not only regulate expression of lipogenic genes, but also affect signalling pathways involved in insulin resistance.

Previous studies have highlighted the importance of short-chain fatty acids (SCFAs) such as acetate, propionate and butyrate in amelioration of chronic inflammatory diseases and promotion of colonocyte health^59^. SCFAs were reported to suppress production of pro-inflammatory cytokines, enhance IL-10 expression and activate Treg cells, leading to amelioration of colitis^60^. SCFAs are produced from fermentation of polysaccharides and some other prebiotics by gut bacteria such as *Bacteroides* spp.^50^ As the percentage of *Bacteroides* was enhanced by treatment of HFD mice with 8% WEGL, it is possible that the amount of SCFAs may also increase in this case. It remains to be determined whether SCFAs are affected or not by WEGL and whether these molecules contribute to the anti-inflammatory effect of WEGL.

Notably, our results show that high molecular weight polysaccharides (>300 kDa) isolated from WEGL produce significant anti-obesity effects in HFD-fed mice. Concerning the possible mechanism of action of polysaccharides from WEGL, previous studies suggest that intestinal bacteria possess distinct polysaccharide preferences and that these molecules may favour the growth of specific bacterial species in the gut microbiota^61^. Collectively, our results suggest that both WEGL and its high molecular weight polysaccharides may be used as prebiotics to reduce body weight gain, chronic inflammation and insulin resistance in obese individuals.

## Methods

### Murine and fungal strains

Animal experiments were approved and performed in accordance with the guidelines of Institutional Animal Care and Use Committee of Chang Gung University (IACUC; Approval No. CGU11-117). Mice of the C57BL/6NCrlBltw genetic lineage were purchased from Biolasco (Taiwan) and kept under controlled light conditions (12 h light–dark cycle), with free access to food and water. Eight-week-old male mice were randomly distributed into six groups containing five to seven animals each (see the legend of Figs 1,4–6,8). Mice were housed in groups of three or four animals per cage, and were fed with either a standard chow diet (13.5% of energy from fat; LabDiet 5001; LabDiet, USA) or a high-fat diet (60% of energy from fat; TestDiet 58Y1; TestDiet, USA). The *G. lucidum* strain initially isolated and characterized at Chang Gung Biotechnology and the water extract of cultured mycelium were processed as described before^62^. Briefly, the water extract was prepared by mixing 400 g of dried *G*. *lucidum* mycelium in 10 l of water before agitation at 150 r.p.m. for 30 min at 121 °C. The solution was cooled to room temperature (22 °C), followed by centrifugation at 5,894*g* for 30 min at 4 °C using a Sorvall RC 3C Plus centrifuge (Thermo Fisher Scientific, USA). The supernatant was concentrated using a Buchi R220 Rotavapor vacuum concentrator (Buchi, Switzerland) at 65 °C to obtain a final volume of 2 l. The extract was sterilized at 121 °C for 20 min, followed by storage at 4 °C in dark glass bottles. The mycelium consisted of <5% crude fibre; >10% polysaccharides; crude proteins at 38.78 g per 100 g; crude fat at 2.41 g per 100 g; carbohydrates at 41.99 g per 100 g; amino acids at 5.2 g per 100 g; and sodium at 76.39 mg per 100 g. Calories were measured at 345 kcal per 100 g. Each group of mice was fed for 2 months with chow diet or HFD, plus daily administration of 100 μl of either water or WEGL at 2, 4 or 8% (w/v) by intragastric gavage. Mean energy intake was assessed by converting the amount of food consumed into calories using the information provided by the manufacturer (LabDiet 5001 and TestDiet 58Y1).

### Preparation of WEGL polysaccharide subfractions

WEGL polysaccharide fractions were provided by Chang Gung Biotechnology. Briefly, 100 ml of WEGL extract was mixed with 600 ml of 95% ethanol (v/v) before incubation for 16 h at 4 °C. The solution was centrifuged at 4,500*g* for 20 min at 4 °C (Sorvall RC 3C Plus centrifuge; Thermo Fisher Scientific, USA). The first supernatant was decanted and 100 ml of 70% cold ethanol was added to produce a pellet. The solution was centrifuged as above and the second supernatant was decanted. This operation was repeated three times. The first and second supernatants were pooled to obtain a volume of 1,050 ml (fraction G4). The precipitate pellet containing crude polysaccharides was mixed with 1,000 ml of distilled water and the material was dissolved completely. The solution of crude polysaccharides was concentrated to a final volume of 700 ml using an R220 vacuum concentrator (Büchi, Switzerland) at 65 °C to remove residual alcohol. Distilled water was added into the crude polysaccharide solution to obtain a final volume of 2,400 ml. The solution was filtered using the Spectrum KrosFlo system with 0.2 μm hollow fibre (1,500 cm^2^, PES; Spectrum Laboratories, USA). The transmembrane pressure was set at 15 p.s.i. A total of 1,800 ml of distilled water was added during filtration of the crude polysaccharide solution. The retentive filtrate (650 ml) was preserved (G1-1). The solutions were filtrated using a 300 kDa cassette membrane (Minimate TFF Capsule; Pall, USA). A total of 600 ml of distilled water was added during filtration. The retentive filtrate (950 ml) was gathered (G1-2). The G1-1 and the G1-2 filtrates were combined to obtain a volume of 1,600 ml (G1). The permeated filtrate was filtrated with a 10 kDa cassette membrane. A total of 600 ml of distilled water was added during filtration. The retentive filtrate (970 ml) (G2) and 3,600 ml of the permeated filtrate (G3) were obtained. The G1, G2, G3 and G4 solutions were concentrated to a final volume of 100 ml using the vacuum concentrator.

### Faecal transplantation

Faecal transplant was performed based on an established protocol^63^. Briefly, 8-week-old male donor mice (*n*=5 per diet group) were fed with Chow, Chow+8% WEGL, HFD or HFD+8% WEGL for 3 months. After 4 weeks of feeding, stools were collected daily for the subsequent 2 months under a laminar flow hood in sterile conditions. Stools from donor mice of each diet group were pooled and 100 mg was resuspended in 1 ml of sterile saline. The solution was vigorously mixed for 10 s using a benchtop vortex (Vortex-Genie 2, Scientific Industries, USA; speed 9), before centrifugation at 800*g* for 3 min. The supernatant was collected and used as transplant material as described below. Fresh transplant material was prepared on the same day of transplantation within 10 min before oral gavage to prevent changes in bacterial composition. Eight-week-old male recipient mice (*n*=5 for each transplant group) were fed with HFD and inoculated daily with fresh transplant material (100 μl for each mouse) by oral gavage for 2 months, before being killed for subsequent analysis. Analysis of body weight and gut microbiota of the donor mice (Supplementary Fig. 9a,b) indicated that the 4-week-long diet treatments used for this cohort of mice produced changes similar to those of the 2-month-long treatments (Figs 1a and 4c).

### Antibodies

Antibodies against IκB-α (nuclear factor of kappa light polypeptide gene enhancer in B cells inhibitor alpha; 1:1,000; 9242L), JNK (c-Jun N-terminal; 1:1,000; 9252L), phosphorylated JNK (1:1,000; 4668S), phosphorylated IRS-1 (insulin receptor substrate-1; phosphorylation on serine 307; 1:1,000; 2381S), IRS-1 (1:1,000; 2382L), Akt (1:1,000; 4691L), phosphorylated Akt (phosphorylation on serine 473; 1:1,000; 4060L) and TLR4 (1:1,000; 2219) were purchased from Cell Signaling Technology (USA). Antibodies against zonula occludens-1 (ZO-1; 1:250; 61–7,300) and occludin (1:250; 71–1,500) were obtained from Invitrogen (USA). Secondary antibodies (anti-rabbit IgG, H+L; 1:10,000 and 1:2,000 for primary antibodies from Cell Signaling Technology and Invitrogen, respectively; 111-035-003) were purchased from Jackson ImmunoResearch (USA). Antibody against β-actin (1:20,000; NB600-501) and secondary antibody anti-mouse IgG (1:20,000; sc-2005) were purchased from Novus Biologicals (USA) and Santa Cruz Biotechnology (USA), respectively. FITC-conjugated anti-F4/80 (1:100; 11-4801; eBioscience, USA), PE-conjugated anti-CD11b (1:100; 557397; BD Pharmingen, USA), anti-CD11c (1:100; 557401; BD Pharmingen), PE-conjugated anti-CD4 (1:100; 557308; BD Pharmingen), PerCp-Cy5.5-conjugated anti-CD25 (1:100; 551071; BD Pharmingen) and Alexa Flour 488-conjugated anti-Foxp3 (1:100; 560403; BD Pharmingen) antibodies were also used.

### Determination of energy and fat in faeces

Measurements were made based on a protocol reported previously^64^. The energy density was determined using an adiabatic bomb calorimeter (Gallenkamp, UK). Total faecal lipids were extracted as described previously^65^. Briefly, dried faecal samples were homogenized once with heptane/diethylether/ethanol (1:1:1, vol/vol) and twice with heptane/diethylether/ethanol/water (1:1:1:1, vol/vol). Supernatants were collected in glass vials that had been weighed beforehand. Total lipid amount in each supernatant was measured gravimetrically after solvent evaporation and was expressed as a percentage of the weight of the starting faecal sample.

### Oil red O staining

Frozen liver sections (6-μm thick) were stained with Oil Red O (Sigma, USA) for 20 min, and then counter-stained with haematoxylin for 1 min. Sections were examined under light microscopy. A total of 20 tissue sections were analysed for each animal.

### Oral glucose tolerance test

Overnight-fasted mice were given glucose by oral gavage (3 g kg^−1^, 66% solution), and measurement of homeostatic model assessment-insulin resistance was performed as described before^20^. Blood glucose was determined with a glucose meter (Roche Diagnostics, Switzerland) using blood collected from the tip of the tail vein.

### Biochemical analyses

Serum LPS quantification was performed using a commercial kit based on the use of a Limulus amaebocyte extract (Lal kit; Cambrex Bio Science, USA). Samples were diluted 1:20 and heated for 10 min at 70 °C. Serum insulin concentrations were determined in 5 μl of serum using a commercial ELISA kit (Mercodia, Sweden) based on the manufacturer’s instructions. Serum FFA were determined using a commercial detection kit (Biovision, USA).

### Quantitative real-time reverse-transcription PCR

Total RNA was isolated using a total tissue RNA isolation kit (Geneaid, Taiwan). Equal amounts of total RNA were used to synthesize cDNA with the Quant II fast RT kit (Tools, Taiwan). Quantitative real-time reverse-transcription PCR (qRT–PCR) was performed in triplicate using SYBR Green, 384-well plates and the LightCycler 480 Real-Time PCR System (Roche Diagnostics). Each well was loaded with a total of 10 μl containing 1.5 μl of cDNA, 1 μl of target primers, 1.5 μl of water and 5 μl of Kapa SYBR Fast Master Mix. Hot-start PCR was performed for 50 cycles, with each cycle consisting of denaturation for 15 s at 94 °C, annealing for 30 s at 60 °C and elongation for 30 s at 72 °C. The Roche LightCycler software (version 1.5.0, Roche Diagnostics) was used for data analysis. Relative quantification was done using the 2^−ΔΔCT^ method^66^. Expression was normalized against the housekeeping gene glyceraldehyde 3-phosphate dehydrogenase. Mean expression levels of chow-fed mice were set as 100%. The primers used are shown in Supplementary Table 1.

### Western blotting

One hundred mg of adipose, liver or intestine tissue were homogenized in a commercial Pro-Prep Protein Extraction Solution (Intron Biotechnology, South Korea). Total protein lysates were fractionated on a 10% sodium dodecyl sulfate–polyacrylamide gel and electro-blotted onto polyvinylidene difluoride membranes (Immobilon TM-P; Millipore, USA). Membranes were blocked with 5% non-fat milk for 1 h at room temperature in TBST buffer (Tris 10 mM, NaCl 150 mM, pH 7.6, 0.1% Tween 20) and probed with primary antibodies overnight at 4 °C. Membranes were then incubated with horseradish peroxidase-conjugated secondary antibody. The dilutions of primary and secondary antibodies were described in the Antibody section above. Protein bands were developed using enhanced chemiluminescence (Millipore). The original immunoblots were provided in Supplementary Fig. 14.

### Morphometry analysis of epididymal adipose tissues

Freshly isolated epididymal adipose tissues from wild-type or WEGL-treated mice were fixed overnight in 10% formalin, followed by dehydration, embedding in paraffin, and sectioning. Sections of 8 μm were stained with haematoxylin and eosin, and cell size was analysed using the Image J software (National Institutes of Health, USA).

### Flow cytometry analysis

Epididymal adipose and hepatic tissues were rinsed in saline, minced into fine pieces and digested with collagenase (Sigma, USA) in Krebs–Henseleit–HEPES buffer (Sigma, USA; pH 7.4) supplemented with 20 mg ml^−1^ of BSA and 2 mM glucose. Incubation was performed at 37 °C in a shaker for 45 min. The samples were passed through a mesh and fractionated by brief centrifugation (1,000*g*). The pellets or the floating cells were collected to obtain the stromal–vascular fraction or adipocytes in adipose tissues, respectively. Cells in the stromal–vascular fraction and liver fractions were lysed in Pharm Lyse buffer for 30 min at 4 °C and resuspended in Pharmingen stain buffer (Becton Dickinson, USA). Cells were incubated with the commercial antibody 2.4G2, which reacts against the FcgII and FcgIII murine receptors (Becton Dickinson, USA) for 10 min and then with primary antibodies or the matching control isotypes for 30 min at 4 °C. The cells were rinsed twice and resuspended in Pharmingen stain buffer. Flow cytometry analysis was performed using a FACSCalibur (Becton Dickinson, USA). Data analysis was performed using the Kaluza flow cytometry analysis software (Beckman Coulter, USA). Macrophages were identified either as F4/80 and CD11c double-positive cells in adipose tissues or as F4/80 and CD11b double-positive in the liver. CD4, CD25 and transcription factor Foxp3 were analysed for Treg cells.

### Cytokine measurements

IL-1β, IL-6 and TNF-α protein levels were measured using commercial ELISA kits (R&D Systems, USA).

### Gut microbiota analysis

Stool samples were snap-frozen in liquid nitrogen before storage at −80 °C. DNA was extracted using a faecal DNA isolation kit (MoBio Laboratories, USA). For each caecal stool sample, the 16S rRNA gene comprising V3–V5 regions was amplified using a composite forward primer and a reverse primer containing a unique 10-base barcode to tag each PCR product. For each sample, a 50 μl PCR mix was prepared containing 25 ng DNA template, 5 × KAPA HiFi Buffer, 10 mM KAPA dNTP Mix, 1 U μl^−1^ KAPA HiFi DNA Polymerase (KAPA Biosystems, USA) and 0.3 μM of composite primer pairs. The PCR reaction conditions consisted of 95 °C for 3 min, followed by 15–25 cycles of 98 °C for 20 s, 45 °C for 15 s and 72 °C for 15 s, and a final extension of 72 °C for 1 min. The composite primer pairs consisted of the forward primer (5′-GCCTTGCCAGCCCGCTC*ACTCCTACGGGAGGCAGCAG*-3′)—a composite of 454 primer B (underlined) and the universal bacterial primer 338F (italics)—and the reverse primer (5′-GCCTCCCTCGCGCCATCAGNNNNNNNNNN*CCGTCAATTCMTTTGAGTTT*-3′)—a composite of 454 primer A (underlined), a unique 10-base barcode (NNNNNNNNNN) and the broad-range bacterial primer 907R (italics). Replicate PCRs were pooled and amplicons were purified using the QiaQuick PCR Purification Kit (Qiagen, USA). PCR products were sequenced on a 454 FLX pyrosequencer platform according to 454 Roche recommended procedures. High-quality reads for bioinformatics analysis were selected and all of the effective reads from all samples were clustered into OTUs based on 97% sequence similarity according to 454 SOP^67^. Briefly, quality control criteria for selecting raw reads of high quality included removal of sequences that lacked V3–V5 primers or barcode sequence (barcode sequence poorly matched or was not identified) as well as sequences that contained a short variable region (<90 bp) or undetermined bases (>2 bases) in the V3–V5 variable region. De-noising criteria comprised (1) using a 50-bp sliding window to reduce sequencing error and (2) an average quality score of 35 within that window for sequence trimming. All high-quality sequences were aligned using the nearest alignment space termination multi-aligner in SILVA-compatible database alignment. Reads that did not align to the anticipated region of the reference alignment were removed. Chimera sequences identified using the UCHIME algorithm^68^ were removed. All reads were classified using a Bayesian classifier with homemade RDP database. Reads that could not be classified at the kingdom level were removed. Alpha diversity analysis including rarefaction analysis and Shannon index were calculated using QIIME^69^. Fast UniFrac PCoA was performed with the phylogenetic tree constructed by inserting the representative of each OTU also generated using QIIME^69^. According to published statistical methods^70^, the statistical significance of the separation among animal groups in PCoA scores plots was assessed by multivariate analysis of variance test with differences in physiological and biochemical values for statistical significance. Normally distributed data were analysed by analysis of variance (ANOVA) using Tukey’s *post hoc* test (SPSS, USA). The relative abundance of each OTU (log_10_-transformed) was used to construct RDA models and OTUs that were different between animal groups were assessed with Canoco for Windows 4.5 (Microcomputer Power, USA) according to the manufacturer’s instructions. Statistical significance was assessed using the Monte Carlo Permutation Procedure with 499 random permutations.

### Determination of bacteria by quantitative real-time PCR

Using bacteria-specific primers described in Supplementary Table 1, abundance of Firmicutes, Bacteroidetes and total bacteria in faecal stool from each diet group at indicative time point was determined by 2^−ΔΔCT^ method-based quantitative real-time PCR^66^. The relative abundance of Firmicutes and Bacteroidetes was normalized to total bacteria for calculation of Firmicutes-to-Bacteroidetes ratio.

### Statistical analysis

Data obtained from three replicate experiments are shown as means±s.e.m. Differences in body weight were assessed using the unpaired two-tailed Student’s *t*-test. Data sets that involved more than two groups were assessed by one-way ANOVA followed by Newman–Keuls *post hoc* tests (see the legend of Figs 1–3,5,6,8). Next generation sequencing analysis was assessed using Tukey’s honest significant difference *post hoc* tests. A *P* value of 0.05 was considered statistically significant. In the figures, the data with different superscript letters are significantly different based on *post hoc* ANOVA statistical analysis. SPSS version 17.0 was used.

## Additional information

**Accession codes:**Microbiota sequencing data were deposited into NCBI’s Sequence Read Archive database under accession code SRP057860.

**How to cite this article:** Chang, C.-J. *et al.* *Ganoderma lucidum* reduces obesity in mice by modulating the composition of the gut microbiota. *Nat. Commun.* 6:7489 doi: 10.1038/ncomms8489 (2015).

## Supplementary Material

Supplementary Figures and Table Supplementary Figures 1-14, Supplementary Table 1

Supplementary Data 1 Phylogeny and relative abundance of OTUs showing differences between chow- and HFD-fed mice.

Supplementary Data 2 Phylogeny and relative abundance of OTUs showing differences between HFD and HFD+WEGL mice.

Supplementary Data 3 Phylogeny and relative abundance of OTUs altered by WEGL in chow-fed mice.

Supplementary Data 4 Phylogeny and relative abundance of OTUs altered following fecal transplant.

## Figures and Tables

**Table 1:** Molecular weight analysis of polysaccharide subfractions isolated from WEGL mycelium.

**Subfraction**	**Component**	**Molecular weight (MW)**	**Percentage (%)**
G1	Polysaccharide	>300 kDa	33.7
G2	Polysaccharide	10–300 kDa	15.6
G3	Polysaccharide	<10 kDa	4.0
G4	Mono-, di-, oligosaccharide	Undetermined	46.8

Polysaccharides were analysed from a 100-ml solution of 20% WEGL (w/v).

**Table 2:** Monosaccharide composition of WEGL-G1 subfraction (>300 kDa).

	**Man**	**Glc**	**Gal**	**GlcN**	**Ara**	**GalN**	**Rha**	**Fuc**
Concentration (mg l^−1^)	19.16	10.6	6.82	0.44	1.17	ND	0.99	1.18
Percentage (%)	47.5	26.3	16.9	1.1	2.9	ND	2.5	2.9

Ara, arabinose; Fuc, fucose; Gal, galactose; GalN, galactosamine; Glc, glucose; GlcN, glucosamine; Man, mannose; ND, not detected; Rha, rhamnose.
