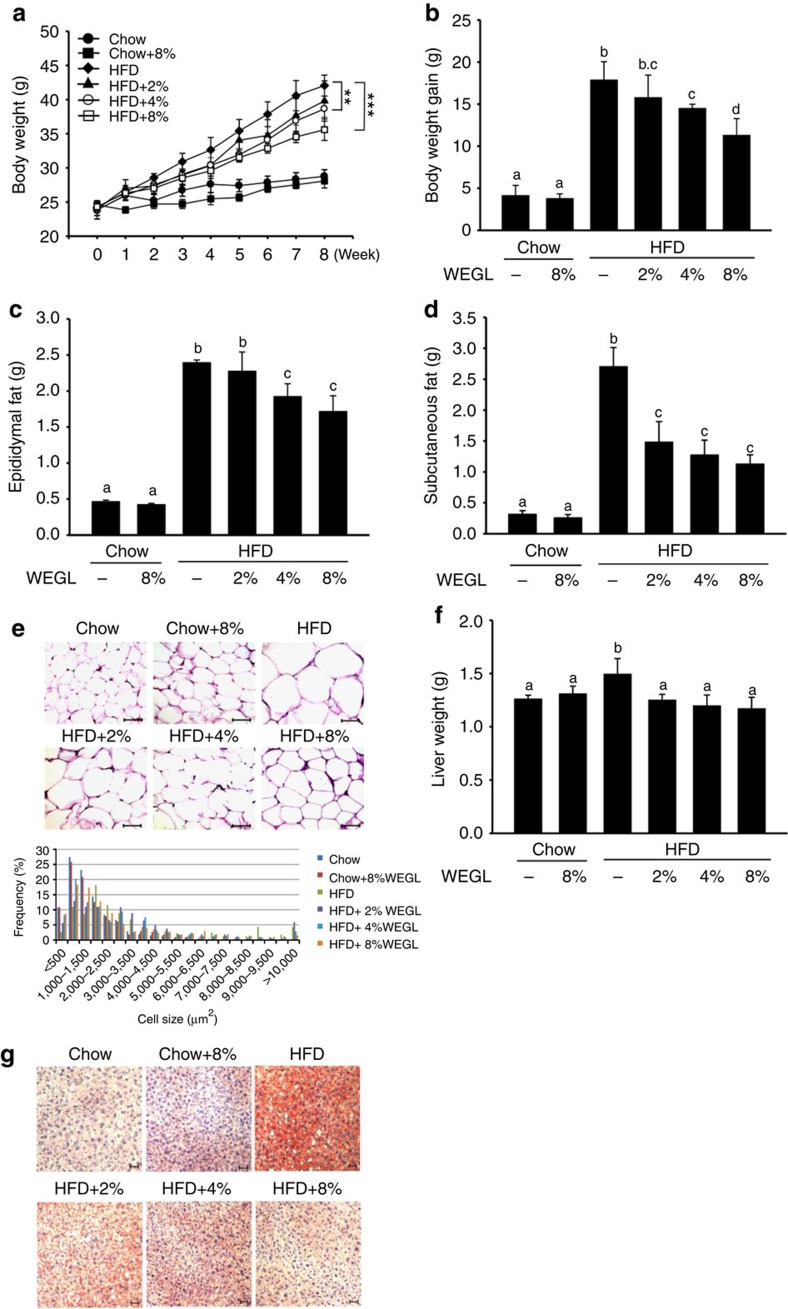# Corrigendum: *Ganoderma lucidum* reduces obesity in mice by modulating the composition of the gut microbiota

**DOI:** 10.1038/ncomms16130

**Published:** 2017-07-11

**Authors:** Chih-Jung Chang, Chuan-Sheng Lin, Chia-Chen Lu, Jan Martel, Yun-Fei Ko, David M Ojcius, Shun-Fu Tseng, Tsung-Ru Wu, Yi-Yuan Margaret Chen, John D Young, Hsin-Chih Lai

Nature Communications 6: Article number:7489; DOI: 10.1038/ncomms8489 (2015); Published: 06 23 2015; Updated: 07 11 2017

The image in Fig. 1e of this Article showing the ‘HFD+8%’ group was inadvertently duplicated from the ‘Chow+8%’ group. The correct version of the figure appears below.

## Figures and Tables

**Figure 1:**