# Supplementary material for: Ganoderma lucidum reduces obesity in mice by modulating the composition of the gut microbiota
Source: Nat Commun. 2015 Jun 23;6:7489. doi: 10.1038/ncomms8489 (PMC4557287; doi:10.1038/ncomms8489)
Supplement: Supplementary Figures and Table — Supplementary Figures 1-14, Supplementary Table 1 [file ncomms8489-s1.pdf]

Supplementary Figure 1 (Lai)

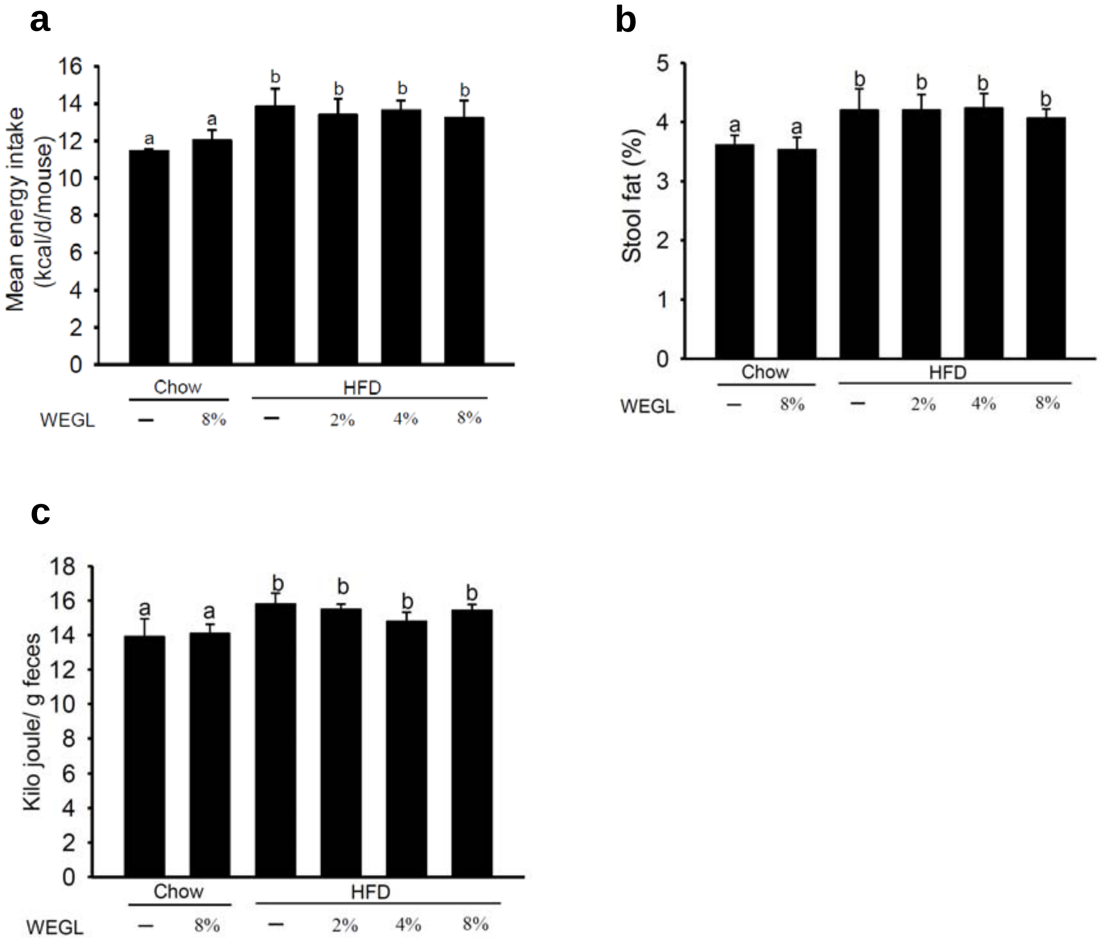

Supplementary Figure 1 | Food intake and energy extraction are not altered by WEGL supplementation. Energy intake (**a**), stool fat (**b**) and energy in feces (**c**) was monitored (n=6 for each group). Energy intake was determined based on calorie intake from consumed food. Fecal lipid and energy were assessed by gravimetric analysis and bomb calorimetry, respectively. Bars with different letters on top represent statistically significant results (P<0.05) based on Newman-Keuls post hoc one-way ANOVA analysis, whereas bars with the same letter correspond to results that show no statistically significant differences.

Supplementary Figure 2 (Lai)

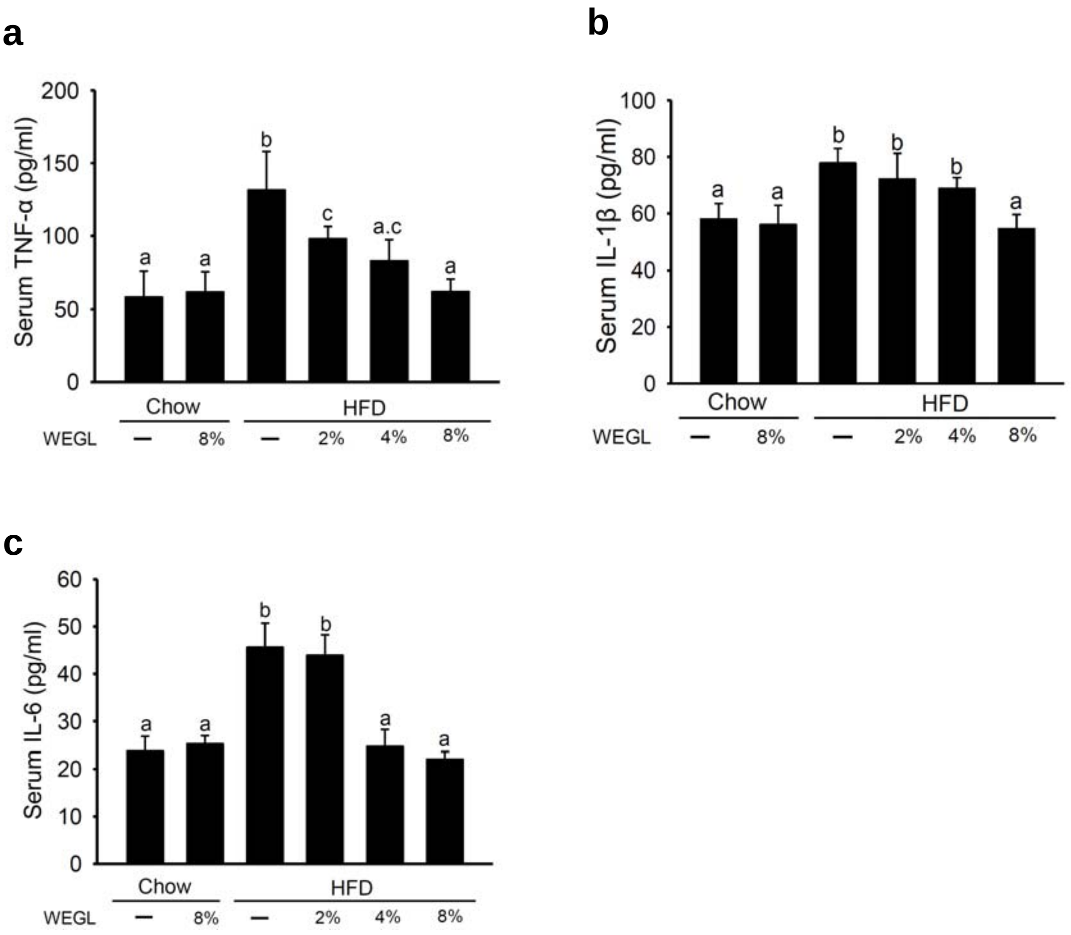

Supplementary Figure 2 | WEGL supplementation reverses HFD-induced production of pro-inflammatory cytokines in serum. Effects of WEGL on production of (**a**) TNF-α, (**b**) IL-1β, and (**c**) IL-6 in serum of chow and HFD mice (n=6 for each group). Bars with different letters on top represent statistically significant results (P<0.05) based on Newman-Keuls post hoc one-way ANOVA analysis, whereas bars with the same letter correspond to results that show no statistically significant differences.

Supplementary Figure 3 (Lai)

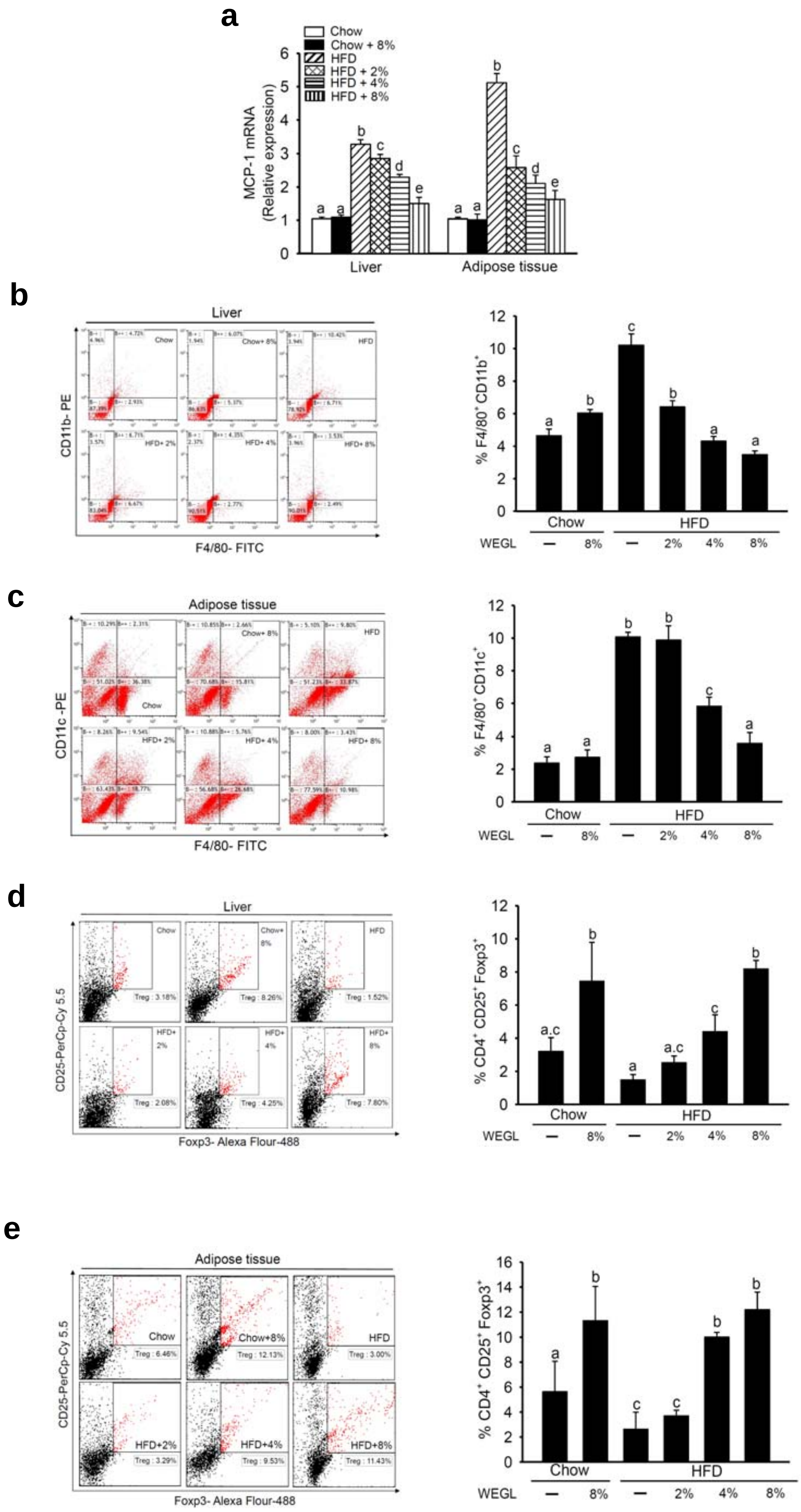

Supplementary Figure 3 | WEGL treatment decreases MCP-1 mRNA expression level and macrophage infiltration in liver and epididymal adipose tissue of HFD-fed mice. Effects of WEGL on MCP-1 mRNA expression in liver and adipose tissues (**a**) and macrophage infiltration in hepatic (**b**) and adipose tissues (**c**) were assessed by qRT-PCR and flow cytometry, respectively. Relative mRNA expression was shown as mean ± SEM in comparison with the Chow group. CD11b-PE (phycoerythrin) and F4/80-FITC (fluorescein-isothiocyanate) were used to stain liver macrophages (Kupffer cells), while F4/80-FITC and CD11c-PE were used to stain macrophages in adipose tissues. Effect of WEGL treatment on Treg cells in hepatic (**d**) and adipose tissue (**e**) indicated by representative plotting of flow cytometry and quantitative analysis (mean ± SEM). Four mice were used per group. Bars with different letters on top represent statistically significant results ($P<0.05$) based on Newman-Keuls post hoc one-way ANOVA analysis, whereas bars with the same letter correspond to results that show no statistically significant differences.

Supplementary Figure 4 (Lai)

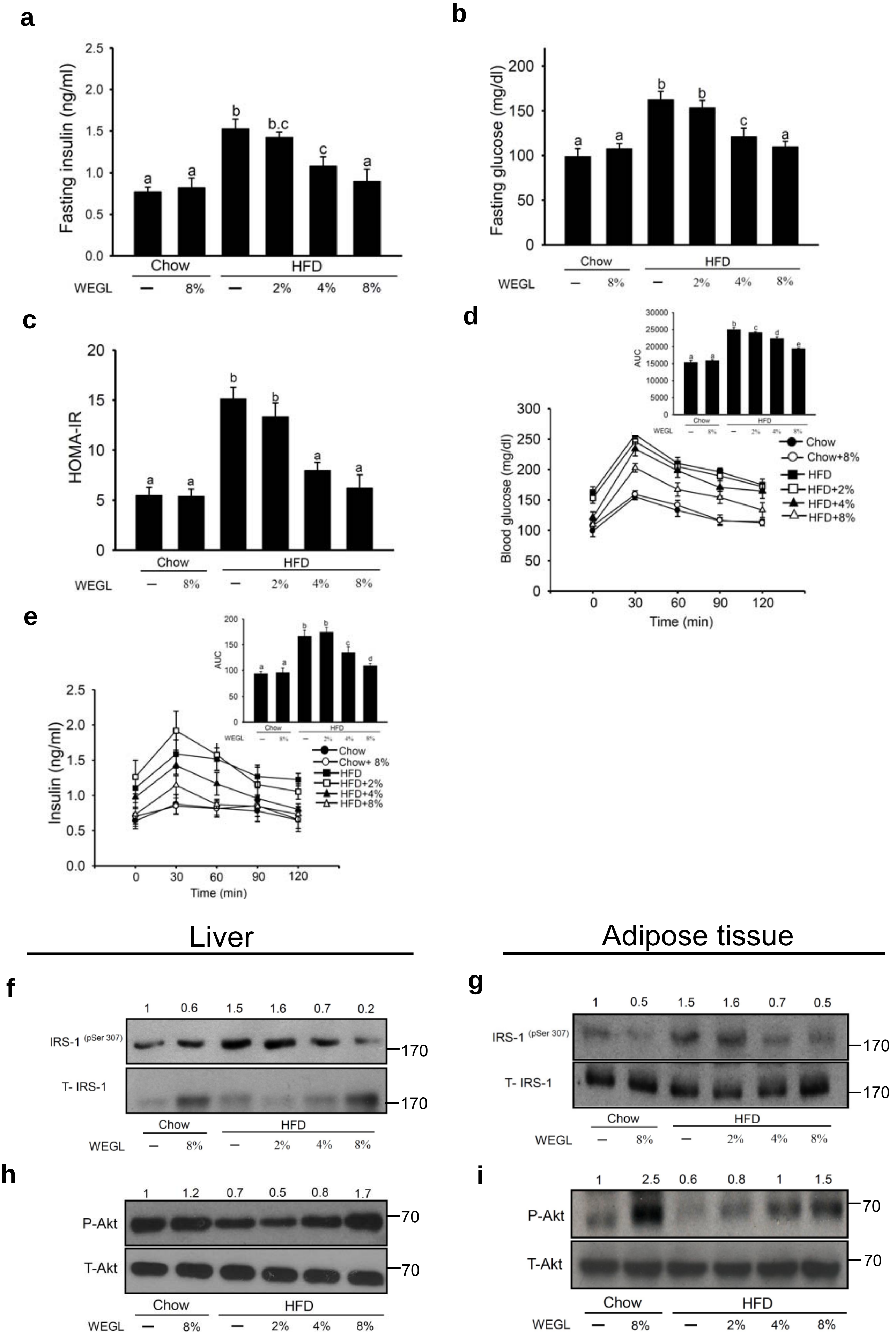

Supplementary Figure 4 | WEGL treatment reduces HFD-induced insulin resistance. Effect of WEGL on fasting insulin (**a**), fasting glucose (**b**), estimated insulin resistance (HOMA-IR) (**c**), Blood glucose (**d**), serum insulin (**e**), IRS-1 phosphorylation (pSer 307) in the liver (**f**) and adipose tissues (**g**), and phosphorylation of Akt in liver (**h**) and adipose tissues (**i**). T-IRS-1 and T-Akt refer to total IRS-1 and Akt, respectively. Insulin and glucose levels were monitored using commercial insulin ELISA kit and a glucose meter, respectively. Area under curve (AUC) in (**d**,**e**) were determined. Representative immunoblots for IRS-1$^{(pSer307)}$, T-IRS-1, P-Akt and T-Akt in hepatic (**f**,**h**) and adipose (**g**,**i**) tissues are shown. Molecular weight markers were indicated as kDa. Protein levels were normalized to internal control (T-IRS-1 or T-Akt) and the relative ratio to Chow group is labeled on the top of immunoblots. Each group consisted of 6 animals. Bars with different letters on top represent statistically significant results ($P<0.05$) based on Newman-Keuls post hoc one-way ANOVA analysis, whereas bars with the same letter correspond to results that show no statistically significant differences.

Supplementary Figure 5 (Lai)

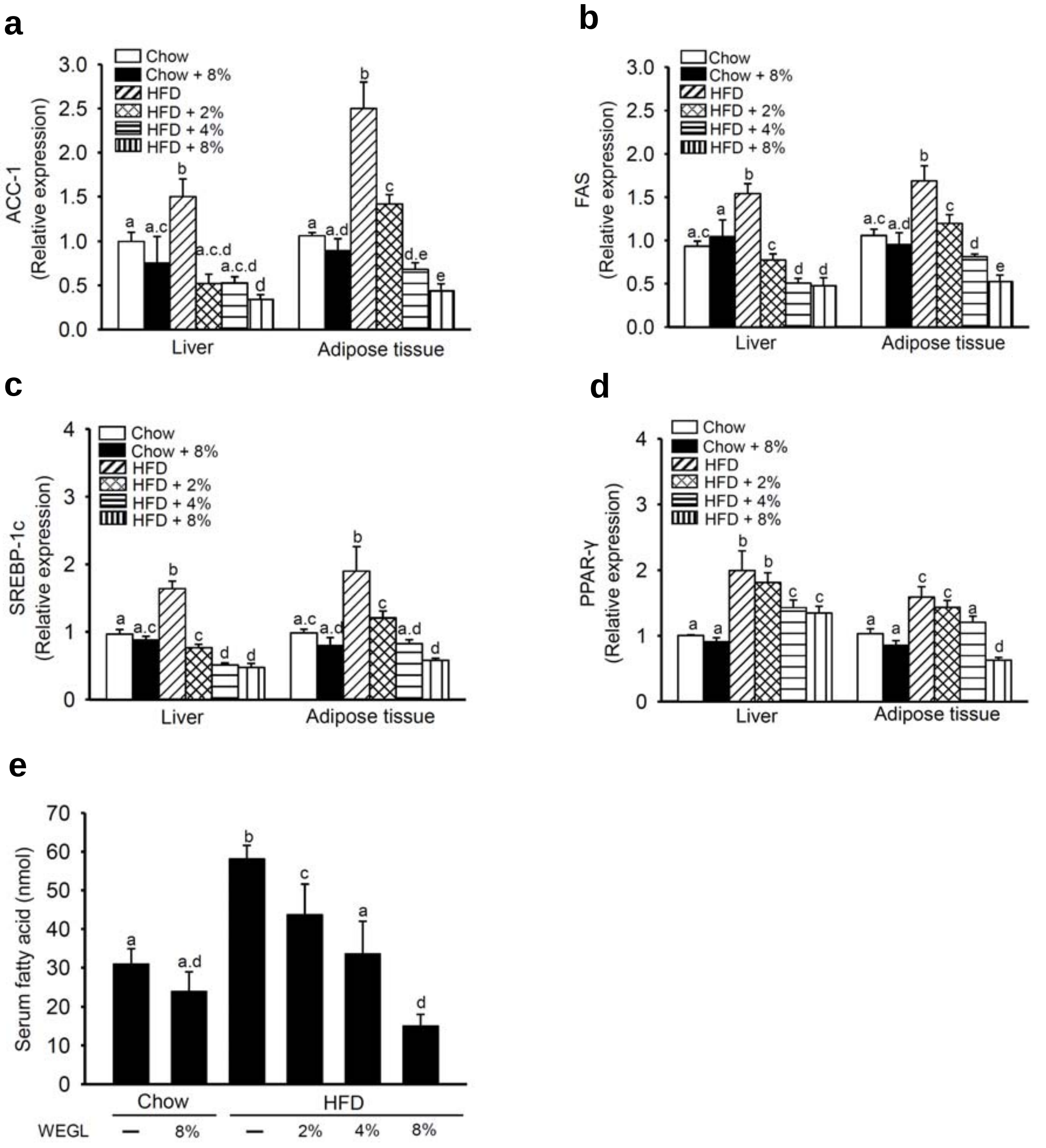

Supplementary Figure 5 | WEGL treatment reverses lipogenic gene expression in HFD-fed mice. Effect of WEGL treatment on mRNA expression of the lipogenic genes ACC-1 (**a**), FAS (**b**), SREBP-1c (**c**), and PPAR- γ (**d**) in hepatic and adipose tissues was monitored using qRT-PCR in comparison with the Chow group. The amount of serum fatty acids (**e**) was also evaluated using commercial FFA detection kit. Results are expressed as mean ± SEM. Each group consisted of 6 animals. Bars with different letters represent statistically significant results (P<0.05) based on Newman-Keuls post hoc one-way ANOVA analysis, whereas bars with the same letter correspond to results that show no statistically significant differences.

Supplementary Figure 6 (Lai)

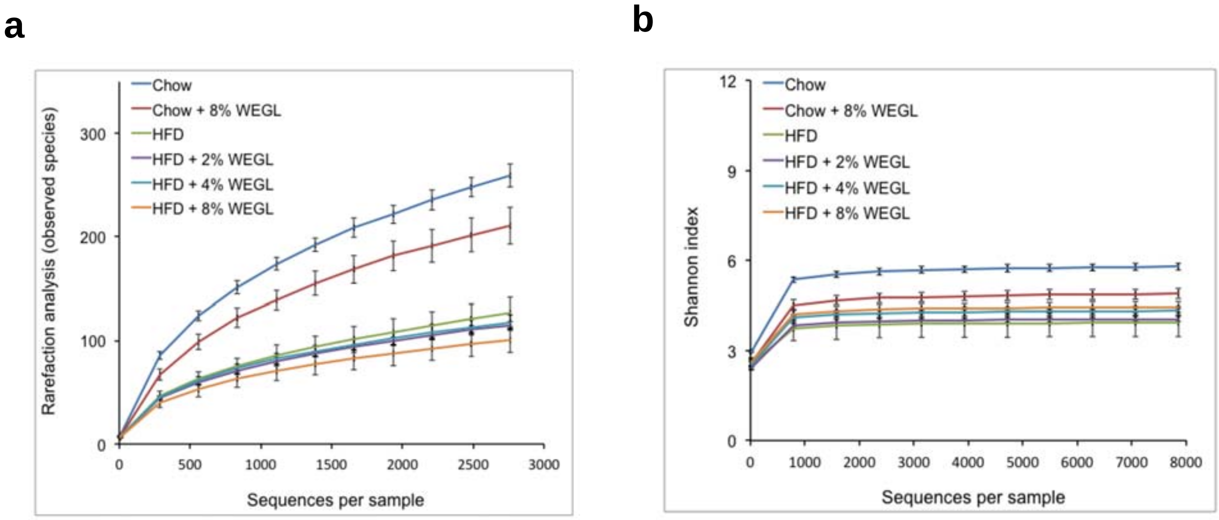

Supplementary Figure 6 | Alpha diversity analysis of WEGL-treated microbiota. Rarefaction analysis (**a**) and Shannon index (**b**) of fecal samples from Chow, Chow + 8% WEGL, HFD, HFD + 2% WEGL, HFD + 4% WEGL, and HFD + 8% WEGL mice (n=7 for each group). Mean ± SEM are shown.

Supplementary Figure 7 (Lai)

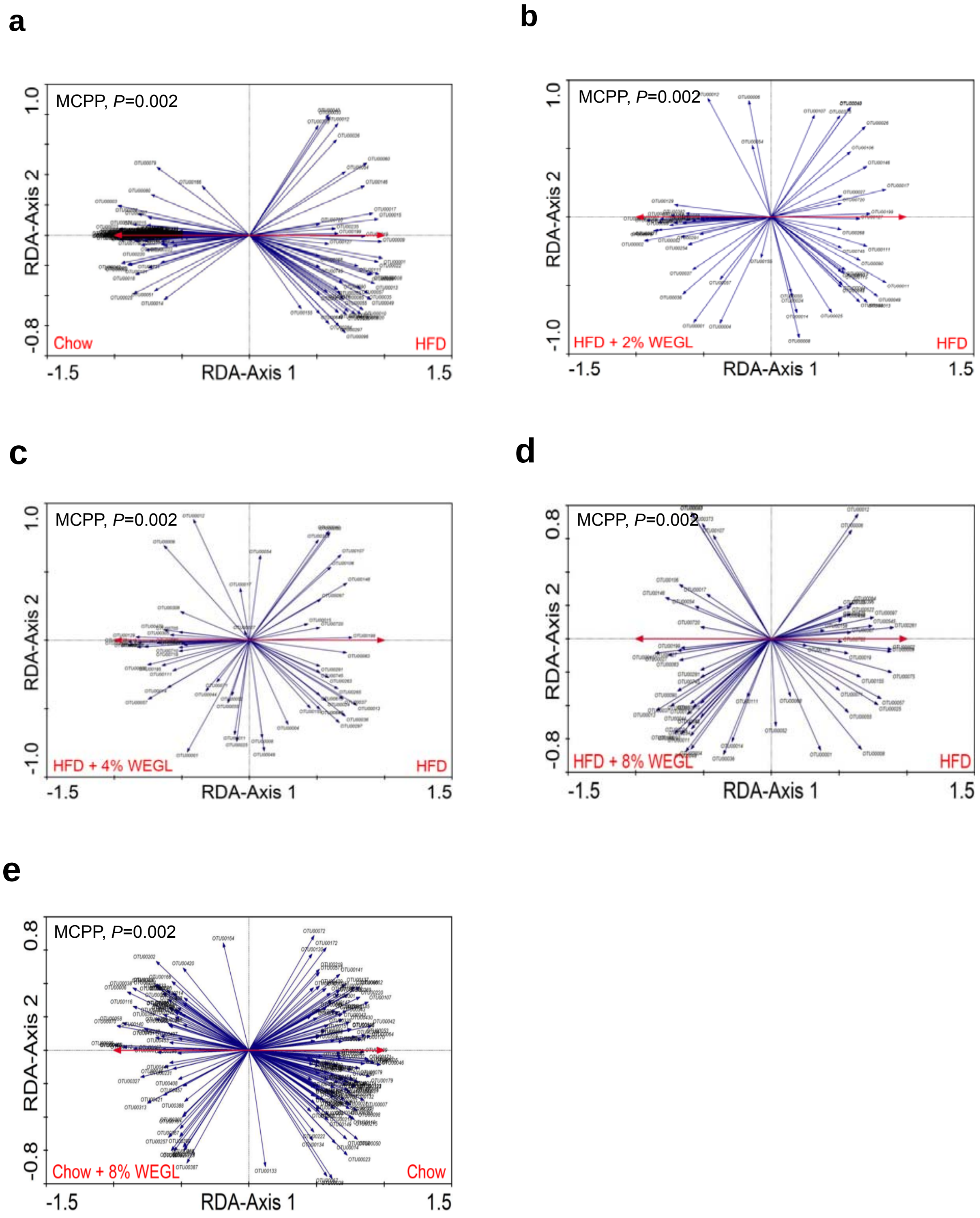

Supplementary Figure 7 | Biplots of altered OTUs from RDA analysis. RDA analysis was performed to compare OTUs of mice fed with chow (**a**), HFD+2% WEGL (**b**), HFD+4% WEGL (**c**), and HFD+8% WEGL (**d**) to OTUs of HFD-fed mice. OTUs of mice fed with Chow+8% WEGL were also compared to OTUs of

chow-fed mice (**e**). OTUs were converted to log_{10}-transformed values. Mouse groups are indicated in red. OTUs that were significantly different (Tukey's HSD test, P<0.05) between the two groups are highlighted by blue arrows. P values calculated with the Monte Carlo permutation procedure (MCPP) are shown in the top left corner.

Supplementary Figure 8 (Lai)

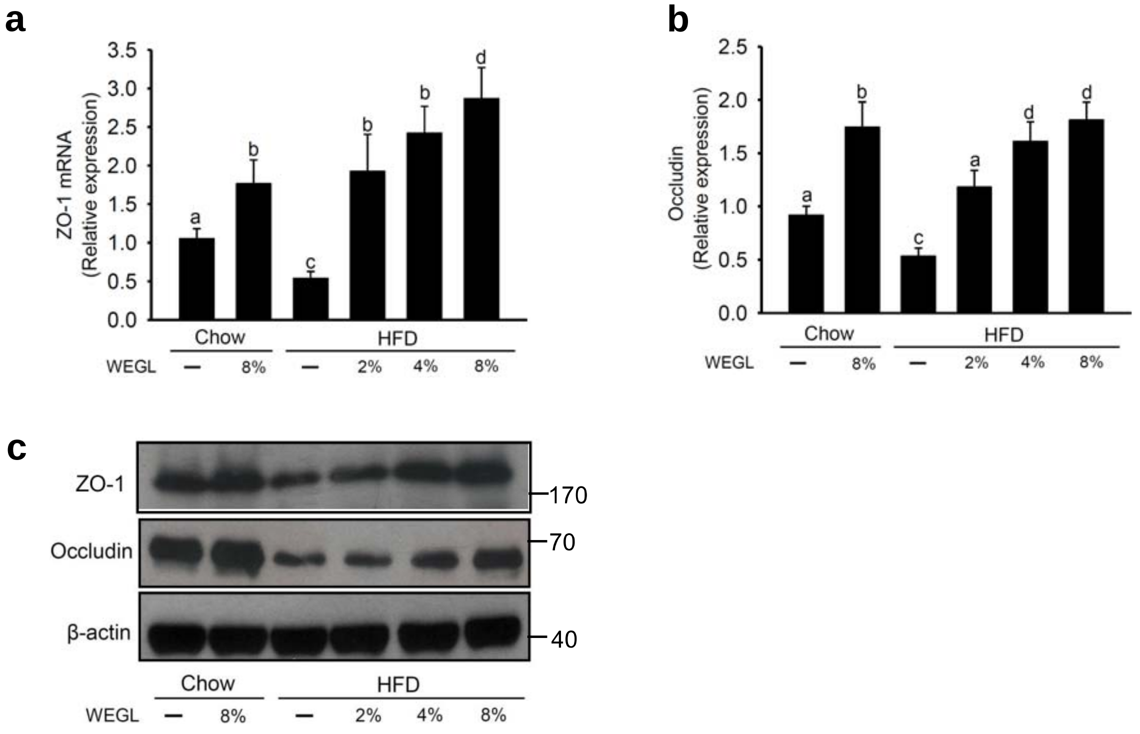

Supplementary Figure 8 | WEGL treatment increases intestinal tight junction expression in HFD-fed mice. Effects of WEGL on mRNA expression levels of ZO-1 (**a**) and occludin (**b**) as well as on the amount of the corresponding proteins (**c**) were determined. Relative mRNA expression in **(a,b)** was shown as mean ± SEM in comparison with the Chow group. Representative ileum immunoblots for occludin, ZO-1, and β-actin in each group **(c)**. Molecular weight markers were indicated as kDa. Each group consisted of 6 animals. Bars with different letters on top represent statistically significant results (P<0.05) based on Newman-Keuls post hoc one-way ANOVA analysis, whereas bars with the same letter correspond to results that show no statistically significant differences.

Supplementary Figure 9 (Lai)

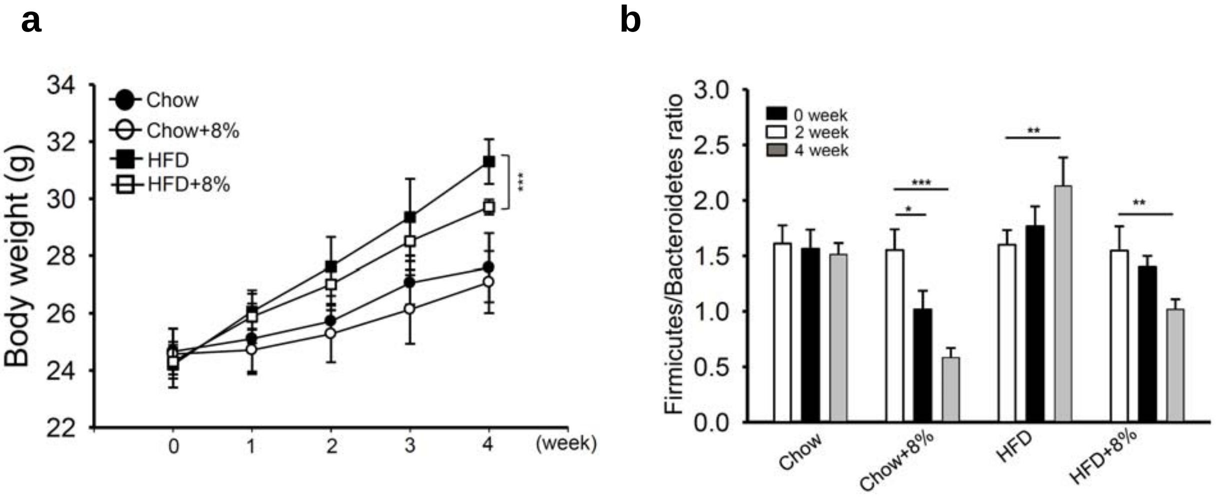

Supplementary Figure 9 | WEGL reduces body weight and Firmicutes-to-Bacteroidetes ratio in the HFD-fed donor mice used for fecal transplantation. Chow- and HFD-fed donor mice were treated daily with 100 μl of either water or 8% WEGL (w/v) by intragastric gavage for one month (n=5 for each group). The effects of WEGL treatment on body weight (**a**) and Firmicutes-to-Bacteroidetes ratio (**b**) in HFD-fed donor mice were determined. Body weight differences in (**a**) were analyzed using unpaired two-tailed Student's *t* test (*** p<0.001). Statistically significant results in (**b**) were determined using Newman-Keuls post hoc one-way ANOVA analysis (* P<0.05, ** P<0.01, *** P<0.001).

Supplementary Figure 10 (Lai)

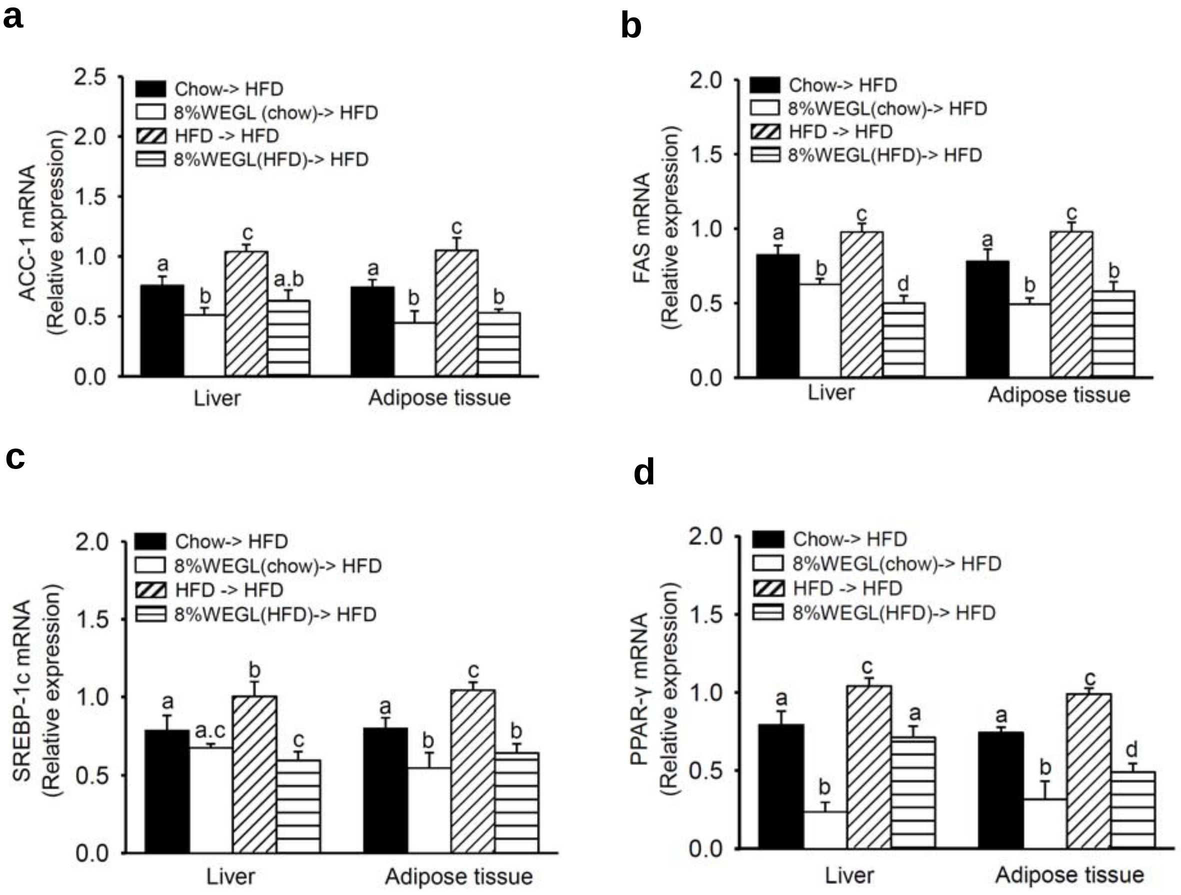

Supplementary Figure 10 | Intestinal microbiota of WEGL-treated obese mice affects lipogenic gene expression. Eight-week-old HFD mice were horizontally transplanted with feces from different mouse groups for two months, followed by measurement of mRNA expression level for ACC-1 (**a**), FAS (**b**), SREBP-1c (**c**), and PPAR- γ (**d**). Relative mRNA expression was shown as mean ± SEM in comparison with HFD→HFD group. Each group consisted of 6 animals. Bars with different letters on top represent statistically significant results ($P<0.05$) based on Newman-Keuls post hoc one-way ANOVA analysis, whereas bars with the same letter correspond to results that show no statistically significant differences.

Supplementary Figure 11 (Lai)

a

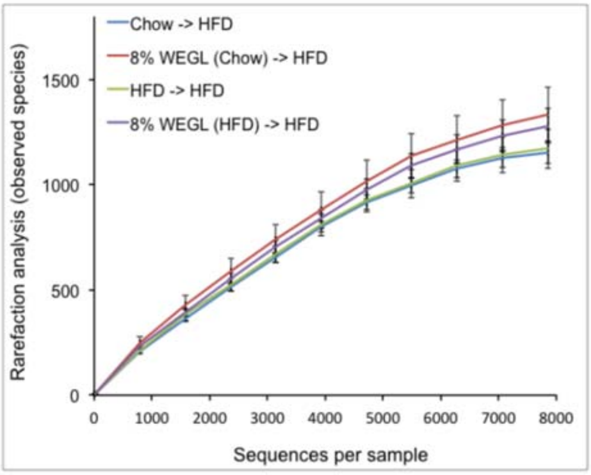

b

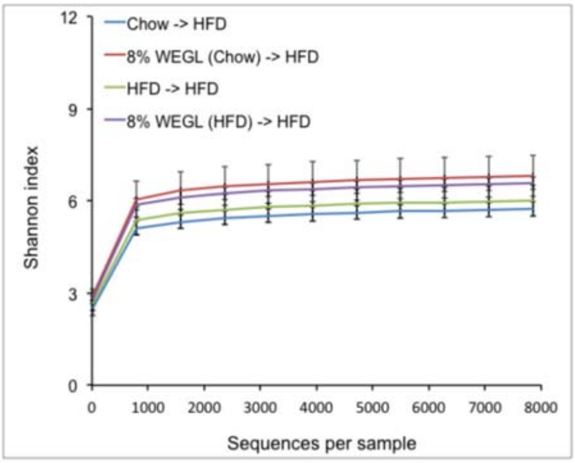

Supplementary Figure 11 | Alpha diversity analysis of microbiota after fecal transplantation. Rarefaction analysis (**a**) and Shannon index (**b**) of fecal samples from Chow→HFD, 8% WEGL (Chow) →HFD, HFD→HFD, and 8% WEGL (HFD) →HFD mice (n=5 for each group). Mean ± SEM are shown.

Supplementary Figure 12 (Lai)

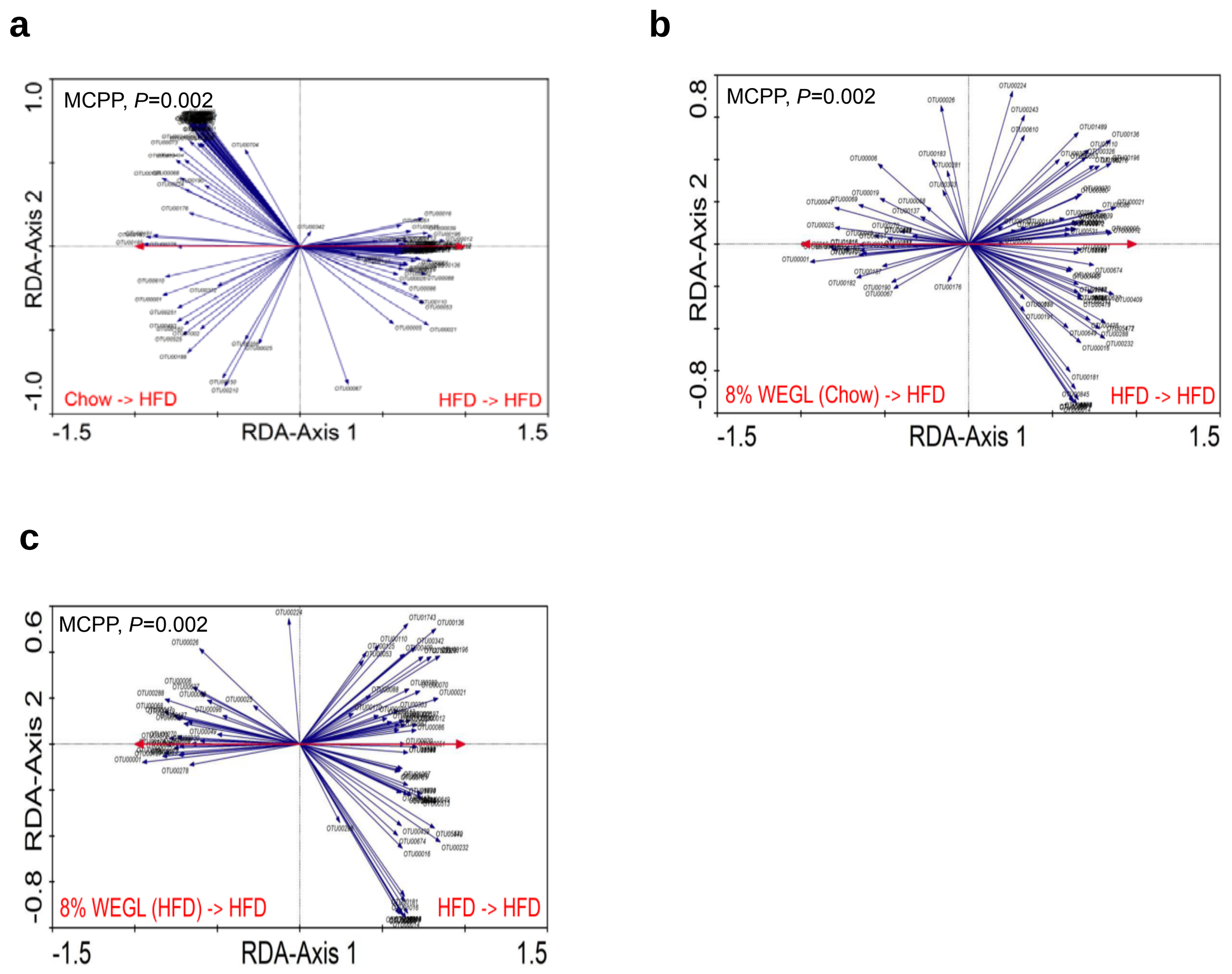

Supplementary Figure 12 | Biplots of altered OTUs from RDA analysis following fecal transfer. RDA analysis showing altered log_{10}-transformed OTUs for fecal transplantation of Chow→HFD (**a**), 8% WEGL (Chow)→HFD (**b**), and 8% WEGL (HFD)→HFD (**c**) compared to HFD→HFD transplanted mice. Mice groups are indicated in red. OTUs that were significantly different (Tukey's HSD test, P<0.05) between two transfer groups are highlighted by blue arrows. P values calculated with the MCPP are shown in the top left corner.

Supplementary Figure 13 (Lai)

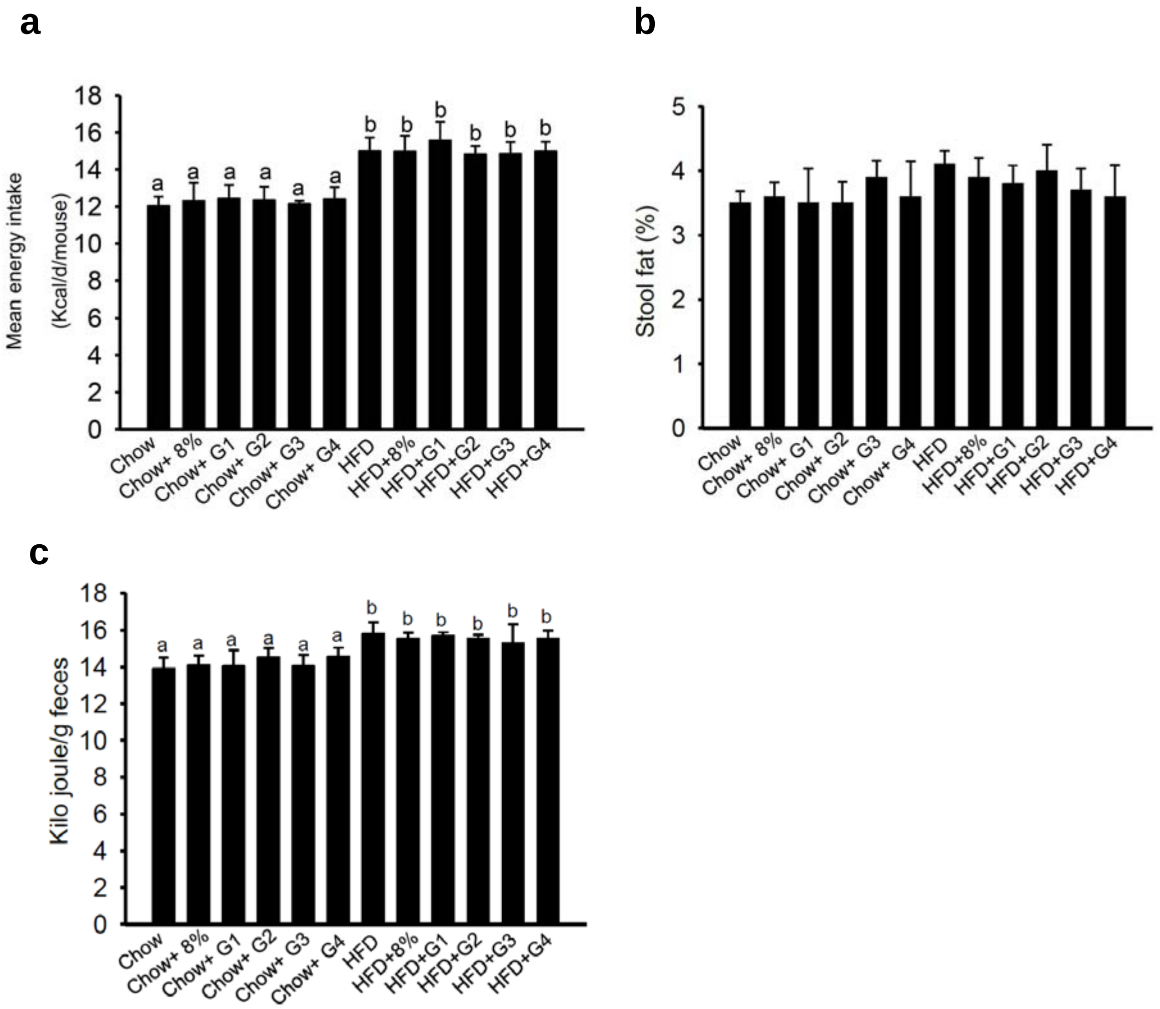

Supplementary Figure 13 | Food intake and energy extraction are not altered by WEGL polysaccharides. Mean energy intake (**a**), stool fat (**b**) and feces energy (**c**) was monitored (n=6 mice for each group). Energy intake was determined based on calorie intake from consumed food. Fecal lipid and energy were assessed by gravimetric analysis and bomb calorimetry, respectively. Bars with different letters on top represent statistically significant results (P<0.05) based on Newman-Keuls post hoc one-way ANOVA analysis, whereas bars with the same letter correspond to results that show no statistically significant differences.

Supplementary Figure 14 (Lai)

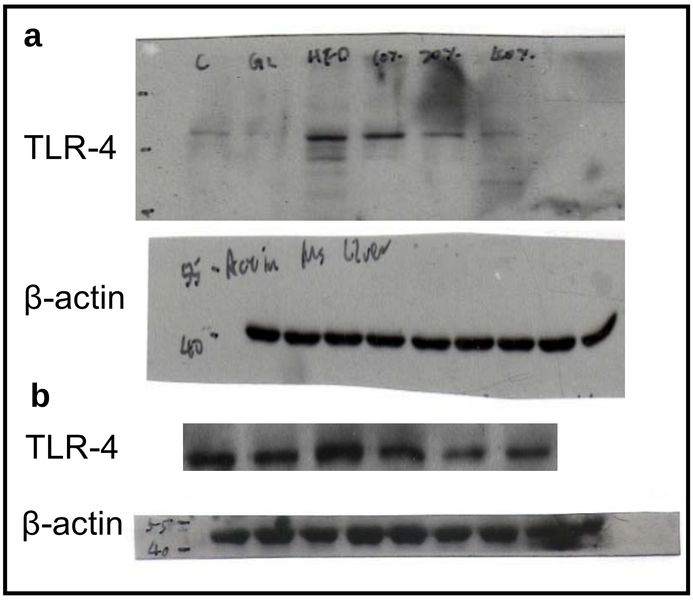

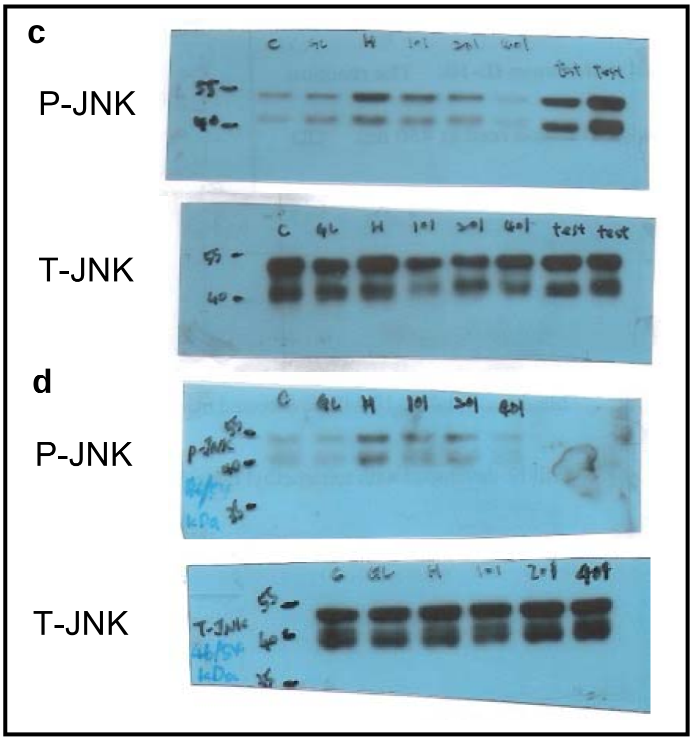

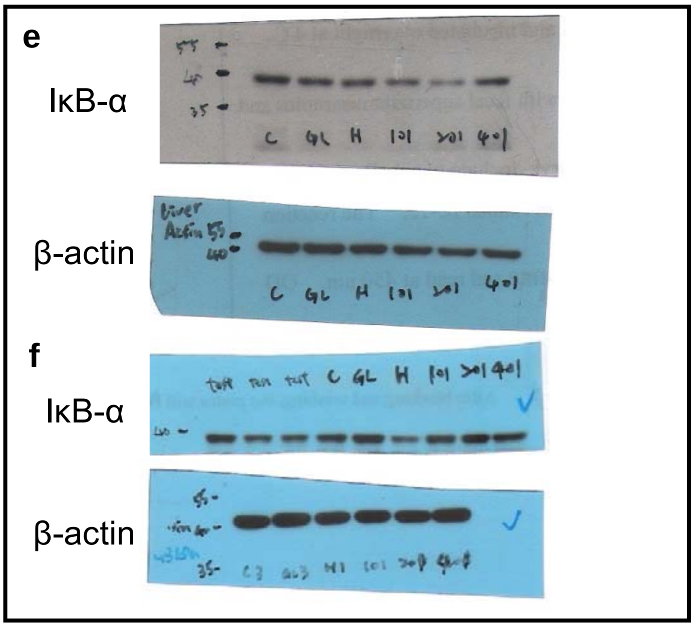
e
IκB-α
β-actin
f
IκB-α
β-actin

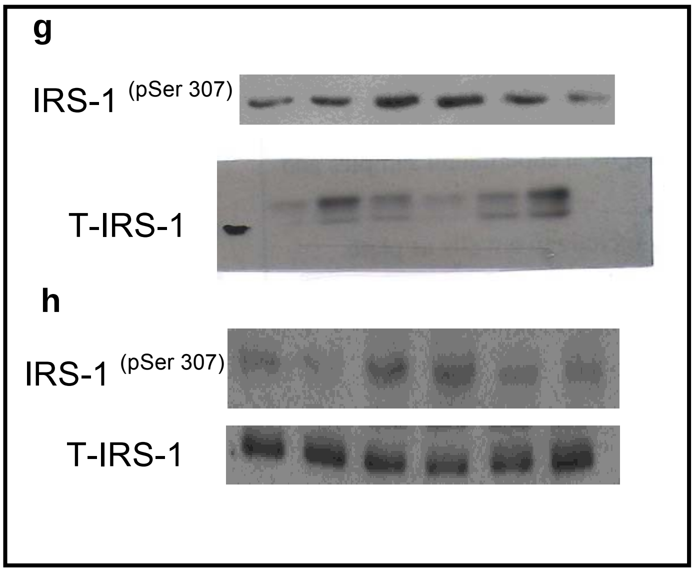
g
IRS-1 (pSer 307)
T-IRS-1
h
IRS-1 (pSer 307)
T-IRS-1

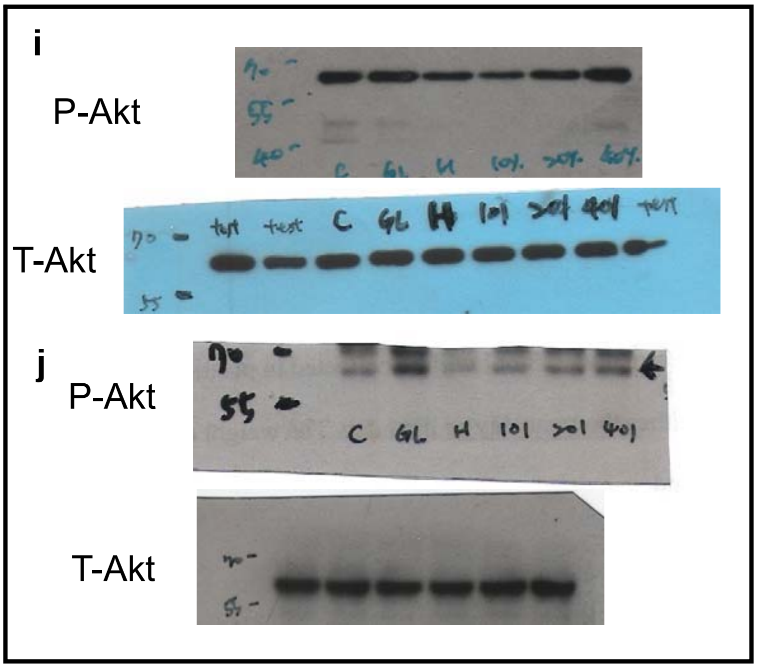

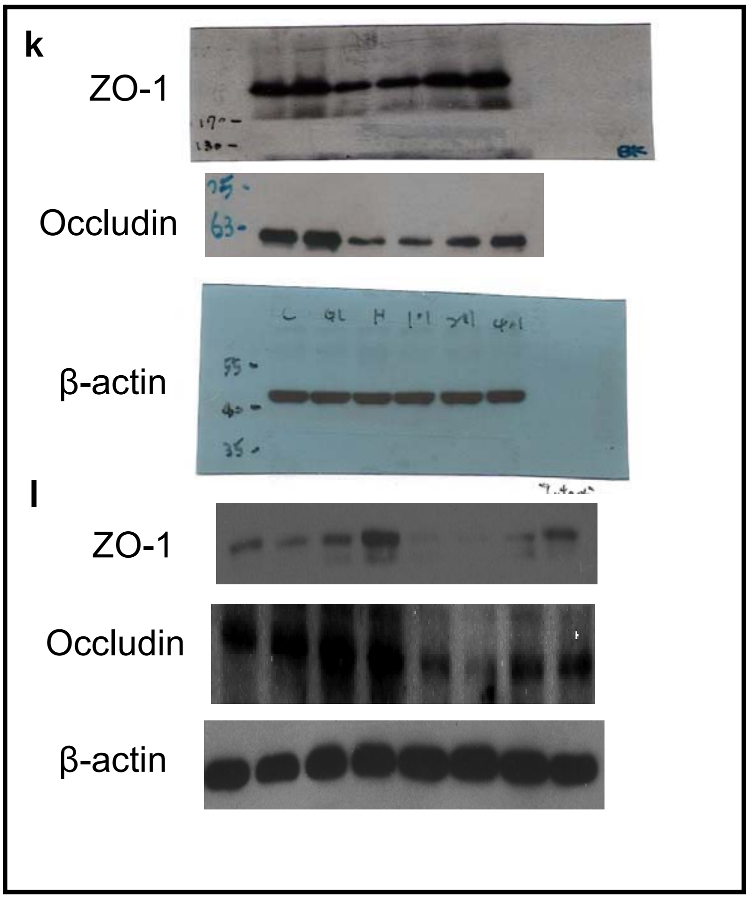

Supplementary Figure 14 | Unedited immunoblots for Fig. 3b (**a**), Fig. 3c (**b**), Fig. 3d (**c**), Fig. 3e (**d**), Fig. 3f (**e**), Fig. 3g (**f**), Supplementary Fig. 4f (**g**), Supplementary Fig. 4g (**h**), Supplementary Fig. 4h (**i**), Supplementary Fig. 4i (**j**), Fig. 6g (**k**) and Supplementary Fig. 8c (**l**)

Supplementary Table 1. Primers used in this study

Name	Sequence
TNF-α Forward	5’-TAGCCAGGAGGGAGAACAGA-3’
TNF-α Reverse	5’-TTTTCTGGAGGGAGATGTGG-3’
IL-6 Forward	5’-CCGGAGAGGAGACTTCAC-3’
IL-6 Reverse	5’-TCCACGATTTCCCAGAGA-3’
IL-1β Forward	5’-TTGAAGAAGAGCCCATCCTC -3’
IL-1β Reverse	5’-CAGCTCATATGGGTCCGAC -3’
IL-10 Forward	5’-GCTCTTACTGACTGGCATGAG -3’
IL-10 Reverse	5’-CGCAGCTCTAGGAGCATGTG-3’
MCP-1 Forward	5’-TCACTGAAGCCAGCTCTCTCT -3’
MCP-1 Reverse	5’-GTGGGGCGTTAACTGCAT-3’
PAI-1 Forward	5’-TCAGCCCTTGCTTGCCTCAT-3’
PAI-1 Reverse	5’-GCATAGCCAGCACCGAGGA-3’
FAS Forward	5’-GCTGCGGAAACTTCAGGAAAT-3’
FAS Reverse	5’-AGAGACGTGTCACTCCTGGACTT-3’
SREBP-1c Forward	5’-GATGTGCGAACTGGACACAG-3’
SREBP-1c Reverse	5’-CATAGGGGGCGTCAAACAG-3’
ACC-1 Forward	5’-GAGTGACTGCCGAAACATCTCTG-3’
ACC-1 Reverse	5’- GCAAGGAGGACAGAGTTTATCGTG-3’
PPAR-γ Forward	5’-GCAGCTACTGCATGTGATCAAGA-3’
PPAR-γ Reverse	5’-GTCAGCGGGTGGGACTTTC-3’
ZO-1 Forward	5’-ACCCGAAACTGATGCTGTGGATAG-3’
ZO-1 Reverse	5’-AAATGGCCGGGCAGAACTTGTGTA-3’
Occludin Forward	5’-ATGTCCGGCCGATGCTCTC-3’
Occludin Reverse	5’-TTTGGCTGCTCTTGGGTCTGTAT-3’
GAPDH Forward	5’-GCATCCACTGGTGCTGCC -3’
GAPDH Reverse	5’-TCATCATACTTGGCAGGTTTC-3’
Total bacteria Forward	5’-ACTCCTACGGGAGGCAGCAG-3’
Total bacteria Reverse	5’-ATTACCGCGGCTGCTGG-3’
Firmicutes Forward	5’-GGAGYATGTGGTTTAATTCGA-3’
Firmicutes Reverse	5’-AGCTGACGACAACCATGCAC-3’
Bacteroidetes Forward	5’-GGARCATGTGGTTTAATTCGATGAT-3’
Bacteroidetes Reverse	5’-AGCTGACGACAACCATGCAG-3’
